# Age-Related Variation in Flavonoid Composition, Human Pancreatic Lipase Inhibition, and Preliminary Brine Shrimp Lethality of *Rhododendron adamsii* Leaves and Sagan Dali Tea

**DOI:** 10.3390/foods15193433

**Published:** 2026-09-25

**Authors:** Daniil N. Olennikov, Nina I. Kashchenko, Nadezhda K. Chirikova

**Affiliations:** 1 Laboratory of Biomedical Research, Institute of General and Experimental Biology, Siberian Division, Russian Academy of Sciences, 6 Sakhyanovoy Street, 670047 Ulan-Ude, Russia; ninkk@mail.ru; 2 Department of Biochemistry and Biotechnology, North-Eastern Federal University, 58 Belinsky Street, 677027 Yakutsk, Russia; hofnung@mail.ru

**Keywords:** *Rhododendron adamsii*, sagan dali, LC–MS, flavonoids, myricetin, quercetin, lipase inhibition, toxicity

## Abstract

*Rhododendron adamsii* Rehder is an important Asian ericaceous species widely used throughout the region as a food plant for producing sagan dali tea. Although the leaves of *R. adamsii* are consumed at different developmental stages, potential variations in their metabolite composition and bioactivity have not yet been scientifically investigated. To address this knowledge gap, first-year, second-year, and litterfall leaves of *R. adamsii*, together with sagan dali tea decoctions, were subjected to LC–MS profiling and quantification, in vitro assays measuring human pancreatic lipase (hPL) inhibition, and toxicity evaluation using the brine shrimp lethality (BSL) assay. The results showed that leaf age significantly affects (*p* < 0.05) phenolic diversity, total content, and antilipase activity in sagan dali tea, establishing the following performance ranking: first-year > second-year > litterfall. Leaf senescence caused a decrease or disappearance of flavonoid glycosides, accompanied by an increase in free aglycones, ultimately altering the tea’s chemical composition. Specifically, the predominant compounds in first-year leaves were myricetin 3′-*O*-xylopyranoside (881–917 mg/100 g) and quercetin 3-*O*-galactoside (672–714 mg/100 g), whereas free myricetin (427–451 mg/100 g) predominated in the litterfall. The highest antilipase potency of sagan dali tea was recorded in first-year leaves (IC_50_ 30.65–33.47 μg/mL vs. orlistat 35.28 μg/mL). This activity was associated with the presence of myricetin, quercetin, and gossypetin in both free and glycosylated forms, with IC_50_ values lower than that of orlistat under the tested conditions. Furthermore, the BSL assay showed LC_50_ values > 1000 μg/mL, indicating low lethality of sagan dali tea to brine shrimp across all categories under the tested conditions. However, these results provide only preliminary information and do not establish food or human safety. In summary, the age of *R. adamsii* leaves is a critical parameter determining phytocomponent concentrations and associated bioactivity while consistently maintaining low brine shrimp lethality under the tested conditions. These findings provide a biochemical basis for further research into the optimal use of *R. adamsii* leaf material.

## 1. Introduction

Plants belonging to the genus *Rhododendron* in the Ericaceae family, which are widely distributed throughout Asia, Europe, and North America, have a long history of human use as valuable sources of traditional medicines and ornamental landscaping [1]. In addition to their medicinal and ornamental applications, various parts of *Rhododendron* species have historically been used in food production as ingredients in herbal teas and as natural spices and colorants [2]. Asian *Rhododendron* species follow this trend because their aromatic leaves and flowers have consistently attracted research and commercial interest in beverage production [3].

Unlike most tea plants, *Rhododendron adamsii* Rehder, or Adam’s rhododendron, is a wild, uncultivated species harvested only from natural populations in its native habitat [4]. The challenge of introducing this species into cultivation stems from its unique biological traits, including its slow growth rate, high sensitivity to substrate composition and altitude, and limited ability to regenerate damaged shoots [5]. Despite these challenges, sagan dali ranks among the top ten North Asian non-caffeinated herbal teas in terms of sales and commercial brand diversity, competing with traditional herbal teas such as willowherb (*Epilobium angustifolium*) [6], meadowsweet (*Filipendula ulmaria*) [7], red raspberry (*Rubus sachalinensis*) [8], prickly rose (*Rosa acicularis*) [9], and wild strawberry (*Fragaria orientalis*) [10]. This strong commercial demand requires in-depth research into the quality and chemical properties of these products, given their growing integration into people’s daily diets. However, comprehensive studies on *R. adamsii* remain limited and underrepresented in the scientific literature.

The *R. adamsii* plant parts used to prepare beverages include the leaves [4], shoots (or leafy shoots) [11], and flowers [5], each representing a distinct type of sagan dali tea. Because leaves are the most accessible plant part and can be harvested without considerably damaging or uprooting the plant, they are the most common form of sagan dali, accounting for more than 90% of the commercial rhododendron tea market. Three main categories of *R. adamsii* leaves are used to produce leaf-based sagan dali tea: first-year leaves, second-year leaves, and litterfall (Figure 1).

The differences between first- and second-year leaves are directly related to their chronological age, reflecting the plant’s distinct growth dynamics. First-year leaves develop on young, current-season shoots. They are soft, up to 2 cm long, pale green to green, and emit a distinct sweet, herbaceous aroma when crushed. In contrast, second-year leaves occur on older shoots and can reach up to 4 cm in length. These older leaves have a leathery texture, a brownish-green or greenish-brown color, and the characteristic sweet, tea-like scent typical of the species. Litterfall forms when second-year leaves drop and overwinter on the ground, giving them a deep brown color and an aroma with earthy tea notes. Each of these three leaf categories has a unique set of organoleptic parameters. Because the plants are harvested in different geographic locations, these distinct characteristics contribute to the wide variety of commercial sagan dali tea products available on the market.

Plants frequently exhibit substantial chemical differences in tissue composition at different physiological ages, resulting from the accumulation, utilization, and transformation of metabolites during maturation [12]. Therefore, harvesting plants at different growth stages directly affects the chemical profiles of the resulting samples [13]. In food and medicinal plants, these differences influence both flavor characteristics and biological activity, ultimately determining the commercial demand for and quality standards of specific raw materials. Research on *Camellia sinensis* (green tea) has shown that theanine and caffeine levels decline as leaves age [14], along with reductions in total polyphenol content [15] and antioxidant parameters [16,17]. In contrast, catechin concentrations [14], as well as free amino acid and soluble sugar levels [18], increase from the first to the second year of leaf development.

Flavonoids represent the most diverse group of metabolites identified in *R. adamsii*, with approximately 100 compounds reported to date, including derivatives of quercetin, kaempferol, and myricetin, as well as their dihydro analogs, such as taxifolin and aromadendrin [4,5,11,19,20,21,22]. Flavonoid retention in *R. adamsii* leaves of different ages has not previously been studied, despite its critical importance for understanding the phytochemical composition of both the raw materials and the resulting herbal infusions. Evaluating these variations provides essential data on the dietary intake of bioactive compounds from sagan dali tea.

Because *R. adamsii* leaves accumulate high concentrations of flavonoids [4,5], these compounds are expected to contribute considerably to the biological effects of sagan dali tea and other *R. adamsii*-derived products. Among these effects, the antioxidant potential, driven primarily by flavonoids, has already been documented for both crude extracts and individual compounds isolated from *R. adamsii* [4,5]. To further investigate the documented biological activities of this plant, evaluating the effects of its extracts on digestive enzymes is of considerable interest. This investigation is consistent with ethnopharmacological reports on the traditional use of *R. adamsii* decoctions for treating disorders of lipid metabolism, such as obesity and atherosclerosis [23]. Therefore, this study aims to assess the potential of these decoctions to inhibit pancreatic lipase, the primary enzyme responsible for lipid breakdown [24], because pancreatic lipase inhibition is considered a therapeutic strategy for managing impaired lipid metabolism [25]. However, it should be noted that in vitro enzyme inhibition does not directly translate to clinical efficacy. Various natural phenolics have been reported to exert mild inhibitory effects [26], and flavonoids represent a promising class of pancreatic lipase inhibitors that have demonstrated in vitro efficacy [27]. Because quercetin, myricetin, and their derivatives have previously been identified as pancreatic lipase inhibitors [28], their documented presence in *R. adamsii* leaves [4,5] suggests that sagan dali tea may exhibit hPL inhibitory activity, which we aimed to investigate in this study.

When evaluating plants of the genus *Rhododendron* as independent food ingredients or raw materials for food production, safety is a critical concern because certain species are known to synthesize toxic compounds [29]. For *R. adamsii*, these phytotoxins have historically included diterpenes, specifically grayanotoxin (andromedotoxin) [30]. Its presence was previously reported in a methanol extract of shoots, identified by infrared (IR) spectroscopy and melting-point analysis following purification with lead acetate. However, subsequent studies using various LC–MS techniques failed to confirm the presence of grayanotoxin in *R. adamsii* samples collected from different regions of Siberia [4,5,11,19,20]. Furthermore, the scientific literature contains no documented cases of poisoning associated with *R. adamsii* leaves, decoctions, or related beverages. Nevertheless, a comprehensive toxicity assessment remains essential and has yet to be performed. A preliminary, non-animal screening of plant toxicity can be performed using the brine shrimp lethality (BSL) assay, which provides reliable baseline information on potential cytotoxic effects [31]. This method is widely used to screen plant extracts [32] and is applied here to analyze *R. adamsii* for the first time. However, it does not replace comprehensive toxicological evaluation in mammalian models and provides only preliminary data on acute lethality.

Therefore, this study aimed to characterize the phenolic profiles and antilipase activity of first-year, second-year, and litterfall *R. adamsii* leaves, as well as sagan dali tea, with particular emphasis on flavonoid retention and a preliminary toxicity assessment of *R. adamsii* decoctions using the BSL assay. We hypothesized that (1) *R. adamsii* leaf age significantly influences flavonoid profiles and content; (2) these differences result in different levels of flavonoid transfer into the aqueous decoction; and (3) the resulting decoctions differ in their human pancreatic lipase (hPL) inhibitory activity and BSL values, with first-year leaves exhibiting the highest potency.

## 2. Materials and Methods

### 2.1. Plant Material

First-year and second-year leaves of *Rhododendron adamsii* Rehder were collected in the Sakha Republic (Tomponsky District) during the flowering stage (May–June) over three consecutive years (2021–2023). Litterfall leaves without visible signs of damage were collected in September (2021–2023) beneath *R. adamsii* bushes in amounts comparable to those of fresh leaves. The species identity was confirmed by Prof. Dr. Pharmacy Tamara A. Aseeva (Institute of General and Experimental Biology, Ulan-Ude, Russia), and voucher specimens (Table S1) were deposited at the Herbarium of the Laboratory of Biomedical Research, IGEB (Ulan-Ude, Russia). In each sampling year, plant material was collected from three geographically distinct locations in the study area (sampling sites 2021-1 … -3, 2022-1 … -3, and 2023-1 … -3) to provide three independent biological replicates (*n* = 3). At each location, 1000 leaves (20 individual leaves per plant from 50 distinct plants) were harvested and pooled into a single composite sample per site to minimize inter-individual variation. The collected specimens were immediately placed in airtight containers containing silica gel and dried within 10 h in a convection oven at 40 °C. After drying, the leaf samples from each collection site were homogenized and divided into five technical replicates (*n* = 5).

### 2.2. Fast Microcolumn High-Performance Chromatography with Photodiode Array and Quadrupole Time-of-Flight Mass Spectrometry Detection (Fast MC-HPLC-PDA-Q-TOF-MS)

Chromatographic analysis was performed using an LC-20 Prominence liquid chromatograph (Shimadzu, Columbia, MD, USA) equipped with an SPD-30AM photodiode array detector (PDA; Shimadzu) and coupled to an LCMS-9050 quadrupole time-of-flight (Q-TOF) mass spectrometer (Shimadzu). Chromatographic separation was performed on a ProntoSIL-120-5-C18 AQ column (50 × 1 mm, 1 μm; Metrohm AG, Herisau, Switzerland). The mobile phase consisted of eluent A (0.1% formic acid (HCOOH) in water) and eluent B (0.1% HCOOH in acetonitrile). The gradient program was as follows: 0.0–3.0 min, 5–100% B; 3.0–4.5 min, 100% B; 4.5–6.0 min, 100–5% B. The injection volume was 1 µL, the flow rate was maintained at 0.2 mL/min, and the column temperature was maintained at 30 °C. Absorption spectra were recorded across the wavelength range of 200–600 nm.

Mass spectrometry (MS) was performed using electrospray ionization in negative mode (ESI^−^). The MS parameters were as follows: an interface temperature of 300 °C, a desolvation line temperature of 250 °C, and a heating block temperature of 400 °C. Nitrogen (N_2_) was used as the nebulizer gas at a flow rate of 3 L/min, while air served as the heater gas at a flow rate of 10 L/min. Collision-induced dissociation (CID) was performed using argon at a pressure of 270 kPa and a flow rate of 0.3 mL/min. The capillary voltage was maintained at 3 kV, and mass spectra were acquired over an *m*/*z* scan range of 100–1900. Metabolites were identified by comparing their retention times (t_R_), UV spectra, and mass spectral fragmentation patterns with those of reference standards, as previously described [21].

Data acquisition and processing were performed using LabSolutions LCMS workstation software (version 2.01, Shimadzu) equipped with an internal LC–MS library. For metabolite quantification, calibration curves were constructed using a series of commercially available reference standards or laboratory-isolated compounds [21,22] at concentrations ranging from 1 to 100 μg/mL. The calibration equations, coefficients of determination (R^2^), limits of detection (LOD), limits of quantification (LOQ), intra-day and inter-day precision, and recovery rates are presented in Table S2. Each standard solution was analyzed in triplicate (*n* = 3) under identical chromatographic conditions [21]. Linear regression analysis and calibration curve generation (concentration versus peak area) were performed using Advanced Grapher software (version 2.2; Alentum Software, Inc., Ramat-Gan, Israel).

### 2.3. Preparation of Plant Extracts

For LC–MS profiling and quantification of *R. adamsii* leaves, a 100 mg aliquot of powdered plant material was extracted with 10 mL of methanol (MeOH) using ultrasonic-assisted extraction (UAE) at 40 °C for 30 min (ultrasonic power: 400 W; frequency: 40 kHz). After extraction, the mixture was centrifuged at 9000× *g* for 15 min. The resulting supernatant was filtered through a 0.22 μm PTFE syringe filter into a 10 mL volumetric flask, and the final volume was adjusted with MeOH. The internal standard (IS), isorhamnetin, was added as a methanolic solution to achieve a final concentration of 10 μg/mL in each analytical sample. Each MeOH extraction series was prepared and analyzed in quintuplicate (*n* = 5).

Leaf decoctions were prepared by boiling 0.5, 1.0, or 2.0 g aliquots of pulverized *R. adamsii* leaves in 100 mL of distilled water for 20 min. After boiling, the mixtures were filtered through cellulose filter paper into a 100 mL volumetric flask, and the final volume was adjusted with distilled water. Each decoction series was prepared and analyzed in quintuplicate (*n* = 5). Flavonoid recovery (%) was calculated as follows: Recovery (%) = (amount of compound in decoction/amount of compound in the corresponding amount of dry leaf material) × 100.

Dry extracts were prepared by freeze-drying leaf decoctions obtained from 20 g of plant material under the abovementioned conditions, yielding loose, brownish, non-hygroscopic powders with a characteristic odor.

Methanol extracts of first-year, second-year, and litterfall leaves from *R. adamsii* were prepared by sonicating a 25 g aliquot of pulverized leaves with 40%, 70%, or 100% MeOH at a 1:15 (*w*/*v*) ratio at 50 °C for 60 min, followed by filtration through cellulose paper and drying under vacuum.

### 2.4. Human Pancreatic Lipase Inhibition

The inhibitory effects of *R. adamsii* extracts, commercially available reference standards, and laboratory-isolated compounds [21,22] on lipase activity were assessed spectrophotometrically, as previously described [33], with modifications. The assay was performed in 0.1 M Tris-HCl buffer (pH 8.0) containing 0.1% (*w*/*v*) Tween 40. Human pancreatic lipase (hPL) (EC 3.1.1.3, 50 U/mg; Lee Biosolutions, Maryland Heights, MO, USA) was used at a final concentration of 0.02 U/mL, and *p*-nitrophenyl stearate (Sigma-Aldrich, St. Louis, MO, USA) was used as the substrate at a final concentration of 0.1 mM. The reaction mixture was incubated at 37 °C for 30 min, and the absorbance was measured at 410 nm. Orlistat (tetrahydrolipstatin; Sigma-Aldrich, St. Louis, MO, USA) was used as the positive control. The extract concentration range was 1–200 μg/mL. The inhibition percentage was calculated as: Inhibition (%) = [(A_control_ − A_sample_)/A_control_] × 100. Blank samples containing all reagents except the enzyme were used to correct for the intrinsic absorbance of polyphenol-rich extracts. Inhibitory activity was expressed as the IC_50_ value (μg/mL), defined as the inhibitor concentration required to achieve 50% inhibition of enzyme activity. These values were determined graphically by plotting the percentage inhibition against the logarithm of the inhibitor concentration. Each bioactivity series was analyzed in quadruplicate (*n* = 4 technical replicates); independent dose–response experiments were performed in triplicate (*n* = 3).

### 2.5. Brine Shrimp Lethality Assay

The acute toxicity of *R. adamsii* extracts was evaluated using the brine shrimp (*Artemia salina*) lethality assay, as previously described [34]. Briefly, *A. salina* cysts (50 mg; Arsal LLC, Barnaul, Russia) were incubated in a 2-L separatory funnel with continuous bottom aeration at 25 °C in 1 L of artificial seawater (Sigma-Aldrich, St. Louis, MO, USA). After 24 h of incubation, 15 mL of a 0.06% (*w*/*v*) yeast suspension was added as a food source, and incubation continued for an additional 48 h. For the toxicity assessment, 10 motile, live *A. salina* larvae (nauplii) were transferred to separate vessels containing 5 mL of artificial seawater supplemented with the test extracts or isolated compounds at different concentrations (1, 10, 100, and 1000 μg/mL). Artificial seawater without extract was used as the negative control. After 24 h of exposure, the surviving larvae were counted under a stereomicroscope. The median lethal concentration (LC_50_) was determined using probit regression analysis [35] by plotting the logarithm (log_10_) of the concentration against Finney’s probit values [36].

### 2.6. Statistical Analysis

Statistical analyses were performed using three-way analysis of variance (ANOVA) with the following factors: leaf category (first-year, second-year, and litterfall), year (2021, 2022, and 2023), and loading level (0.5, 1.0, and 2.0 g/100 mL), using Statistica software (ver. 12.6, Dell, Round Rock, TX, USA). Post hoc comparisons were performed using Fisher’s least significant difference (LSD) test at α = 0.05. When interactions were significant, the data were analyzed separately for each factor level. Statistical significance was set at *p* < 0.05. Data are presented as means ± standard deviations (S.D.). For correlation analysis, Pearson’s r and corresponding *p*-values were calculated. Linear regression analysis and calibration curve construction were performed using Advanced Grapher 2.2 (Alentum Software, Inc., Ramat-Gan, Israel).

## 3. Results

### 3.1. Phenolic Compounds in First-Year, Second-Year, and Litterfall R. adamsii Leaves

LC–MS analysis of leaf extracts from first-year, second-year, and litterfall *R. adamsii* leaves revealed differences in chromatographic profiles owing to age-related changes in leaf tissue (Figure 2, Table 1).

For first- and second-year leaves, these differences were less pronounced, involving the detection of 25 and 24 compounds, respectively. In litterfall leaves, however, the number of detectable compounds decreased to 14, resulting in a change in the chromatogram profile.

LC–MS identification data revealed that all three sample types contained various compounds belonging to the benzoic acid, hydroxycinnamate, and flavonoid groups. The benzoic acids included vanillic acid derivatives in the form of hexosides (comp. **1**–**3**), with one identified as vanillic acid 4-*O*-glucoside (**2**) [38], and the quinic acid ester **5**. The hydroxycinnamates included caffeic acid (**7**) and *trans*-5-*O*-caffeoylquinic acid (**6**) [39]. The remaining 19 compounds were flavonoids belonging to the flavonol and dihydroflavonol groups, with 18 compounds identified at the first level [37] because their presence was confirmed by comparison with reference substances.

The most diverse group of *R. adamsii* leaf flavonoids included flavonols derived from gossypetin (3,5,7,8,3′,4′-hexahydroxyflavone), myricetin (3,5,7,3′,4′,5′-hexahydroxyflavone), 5-*O*-methylmyricetin (3,5,7,3′,4′,5′-hexahydroxyflavone 5-*O*-methyl ester), and quercetin (3,5,7,3′,4′-pentahydroxyflavone) [4,5,21]. Known gossypetins included gossypetin 3-*O*-galactoside (**8**) and gossypetin 3-*O*-arabinopyranoside (rhododeside A, **11**), along with the tentatively identified gossypetin 3-*O*-pentoside **10**, which was isomeric with compound **11**. Gossypetin 3-*O*-arabinoside **11** and gossypetin 3-*O*-pentoside **10** occurred in first- and second-year leaves but disappeared from litterfall leaves, whereas gossypetin galactoside **8** was present in all samples. Myricetin occurred both in free form (**24**) and as glycosides, including myricetin 3-*O*-galactoside (gmelinoside I, **9**), myricetin 3′-*O*-glucoside (cannabiscitrin, **17**), myricetin 3′-*O*-xyloside (**21**), and myricetin 3-*O*-(6″-*O*-benzoyl)-galactoside (adamsoside IV, **23**). Most of the identified myricetins were present in all *R. adamsii* leaf samples, except for gmelinoside I and adamsoside IV, which were absent from the litterfall leaves. The 5-*O*-methyl ester of gmelinoside I (**14**), or 5-*O*-methylmyricetin 3-*O*-galactoside, was the only methylated flavonoid found in first-year, second-year, and litterfall leaves. The remaining flavonols were quercetins, including the aglycone (**25**) and several known glycosides: quercetin 3-*O*-galactoside (hyperoside, **15**), quercetin 3-*O*-glucoside (isoquercitrin, **16**), quercetin 3-*O*-arabinofuranoside (avicularin, **18**), quercetin 3-*O*-arabinopyranoside (guaijaverin, **19**), quercetin 3-*O*-xylopyranoside (reynoutrin, **20**), and quercetin 3-*O*-(6″-*O*-acetyl)-galactoside (**22**). Only the acetyl ester **22** was not detected in the litterfall leaves, whereas most quercetins were characteristic of all leaf samples.

The small group of dihydroflavonols comprised three compounds: taxifolin 3-*O*-glucoside (glucodistylin, **4**), aromadendrin 3-*O*-glucoside (**12**), and taxifolin 3-*O*-arabinopyranoside (**13**). All three were present in every sample, except glucodistylin, which was not detected in litterfall leaves.

The described compounds **1**–**25** have already been reported for *R. adamsii*. However, their occurrence in relation to plant age is presented here for the first time.

### 3.2. Phenolic Compound Content in First-Year and Second-Year Leaves and Litterfall of R. adamsii

Changes in the qualitative composition (HPLC profile) of plant samples always affect the quantitative levels of the compounds identified in the object under study. To determine how this phenomenon manifests in *R. adamsii* leaves of different ages, we examined changes in first-year, second-year, and litterfall leaves collected from 2021 to 2023 (Table 2 and Table S3). Flavonoids were not only the most diverse group of phenolics but also had the highest concentration in *R. adamsii* leaves of different ages, with flavonoid contents reaching 4081–4219 mg/100 g in first-year leaves, 2706–2963 mg/100 g in second-year leaves, and 1043–1107 mg/100 g in litterfall leaves, whereas non-flavonoid contents ranged from 71–115 mg/100 g in first-year leaves to 67–89 mg/100 g in second-year leaves and 0–1 mg/100 g in litterfall leaves.

The percentage of total flavonoids relative to total phenolics ranged from 97.1% to 100% in all samples studied. In the flavonoid group, flavonols contributed more to the total content than dihydroflavonols, reaching 3788–3896 mg/100 g in first-year leaves, 2486–2699 mg/100 g in second-year leaves, and 987–1050 mg/100 g in litterfall leaves, compared with dihydroflavonol contents of 293–323 mg/100 g in first-year leaves, 223–264 mg/100 g in second-year leaves, and 56–60 mg/100 g in litterfall leaves. The different types of flavonols, classified according to the nature of the aglycone, were present in the raw material in decreasing concentrations as follows: myricetin (1753–1822 mg/100 g) > quercetin (1391–1432 mg/100 g) > gossypetin (341–374 mg/100 g) > 5-methylmyricetin (280–292 mg/100 g) in first-year leaves; quercetin (1052–1156 mg/100 g) > myricetin (910–997 mg/100 g) > gossypetin (322–336 mg/100 g) > 5-methylmyricetin (199–211 mg/100 g) in second-year leaves; and myricetin (738–768 mg/100 g) > quercetin (185–211 mg/100 g) > 5-methylmyricetin (40–54 mg/100 g) > gossypetin (22–30 mg/100 g) in litterfall leaves. The glycoside-to-aglycone ratio varied among the samples, amounting to 15.5–17.8:1 for first-year leaves, 6.2–7.5:1 for second-year leaves, and 0.9–1.1:1 for litterfall leaves, indicating a gradual decrease in glycoside concentration and an increase in aglycone levels, from 220–248 mg/100 g in first-year leaves to 527–568 mg/100 g in litterfall leaves.

The predominant compounds in the first-year leaves were myricetin 3′-*O*-xyloside (881–917 mg/100 g), quercetin 3-*O*-galactoside (672–714 mg/100 g), adamsoside IV (521–556 mg/100 g), quercetin 3-*O*-xylopyranoside (358–383 mg/100 g), and gossypetin 3-*O*-galactoside (306–327 mg/100 g). In the second year, the pattern changed, and the five main compounds, listed in descending order, were quercetin 3-*O*-galactoside (510–561 mg/100 g), adamsoside IV (291–346 mg/100 g), quercetin 3-*O*-xylopyranoside (269–309 mg/100 g), gossypetin 3-*O*-galactoside (311–306 mg/100 g), and myricetin (285–302 mg/100 g). In the litterfall leaves, three flavonoids accounted for more than 70% of the phenolic compounds: myricetin (427–451 mg/100 g), quercetin (100–117 mg/100 g), and myricetin 3′-*O*-xyloside (248–286 mg/100 g). Notably, the shift toward lower levels of all glycosides was particularly pronounced for the acylated flavonoids, quercetin 3-*O*-(6″-*O*-acetyl)-galactoside and myricetin 3-*O*-(6″-*O*-benzoyl)-galactoside, which were absent from the litterfall leaves. Overall, the transition from first-year leaves to litterfall leaves was characterized by decreased concentrations of glycosides and esters and increased levels of aglycones.

### 3.3. Transfer of Flavonoids from R. adamsii Leaves to Sagan Dali Tea Decoctions

Although plant material may contain potentially high concentrations of compounds, the level of target metabolites in the finished product may be lower than expected, which in most cases results from the specific characteristics of the production process. A similar phenomenon is often observed in the production of water-based extracts, such as tea beverages, which are sources of various groups of phenolic compounds. In this case, water is not an optimal solvent for most phenolics because they generally have a greater affinity for solvents of medium polarity, such as alcohols and ketones. Flavonoids, which constitute more than 97% of the extractable phenolic compounds in *R. adamsii* leaves, are primarily flavonol and dihydroflavonol glycosides that are efficiently transferred into solvents such as ethanol, methanol, and other alcohols. To evaluate sagan dali tea, it is necessary to determine what proportion of the original flavonoid content in the raw material is transferred to the water during decoction. Given the absence of official recommendations regarding the leaf-to-water ratio, we studied decoctions prepared at three loading levels (i.e., 0.5, 1.0, and 2.0 g per 100 mL of water) using first-year, second-year, and litterfall leaves of *R. adamsii* (Table 3 and Table S4).

Across all sagan dali decoctions, the general trend was higher compound content in the high-loading groups (2.0 g per 100 mL) than in the lowest-loading groups (0.5 g per 100 mL). The leaves’ higher initial flavonoid content resulted in greater transfer into the water and the production of a more concentrated drink. In first-year sagan dali tea with a loading of 2.0 g per 100 mL, the total flavonoid content was 62,803 μg/100 mL, of which 58,610 μg/100 mL consisted of flavonols, including 24,041 μg/100 mL of quercetin derivatives and 27,378 μg/100 mL of myricetin derivatives.

In the same sample, the concentrations of the principal compounds reached 14,637 μg/100 mL for myricetin 3′-*O*-xylopyranoside, 11,562 μg/100 mL for quercetin 3-*O*-galactoside, 6445 μg/100 mL for adamsoside IV, 4988 μg/100 mL for 5-*O*-methylmyricetin 3-*O*-galactoside, and 4066 μg/100 mL for myricetin. The quantitative values for sagan dali in litterfall leaves at a loading of 0.5 g per 100 mL were substantially lower, amounting to 1354 μg/100 mL for the total flavonoid content, which represented approximately 2% of the flavonoid level in the decoction of first-year leaves at a loading of 2.0 g/100 mL. The quantitative data for second-year leaves occupied an intermediate position but were closer to those for first-year leaves.

Calculation of flavonoid recovery as an indicator of the efficiency of compound transfer from leaves to water during boiling revealed no consistent patterns among *R. adamsii* leaves of different ages, loading types, or individual compounds (Table S4). However, the following features can be noted (Figure 3).

First-year leaves exhibited higher recovery of most compounds in the decoctions than did litterfall leaves. Of the 18 compounds identified in first-year leaves, 12 showed >80% recovery under various loading conditions. Only three flavonoids reached the same recovery level in second-year leaves, whereas none reached this level in litterfall leaves, where the maximum recovery was just over 40% for only six compounds. This finding highlighted the need to examine each decoction variant because the same compounds showed different recovery levels when the loading type or plant age changed. Total flavonoid recovery in decoctions of first-year, second-year, and litterfall leaves of *R. adamsii* showed values of 72–77%, 52–59%, and 24–31%, respectively.

### 3.4. Human Lipase-Inhibitory Potential of Sagan Dali Tea Decoctions

The high concentration of flavonoids in water decoctions of *R. adamsii* leaves indicates their potential as inhibitors of digestive enzymes [40], including lipase, whose activity may be reduced by flavonoids that block the enzyme’s active sites and impair its fat-breaking activity [24]. An in vitro study examining the effect of sagan dali tea on lipase used hPL, and its activity was inhibited by nine samples of *R. adamsii* leaf decoctions prepared as dry extracts, as well as by all pure flavonoids identified in these extracts. Freeze-dried decoctions in the form of dry extracts provide a stable form with a constant chemical composition and a high concentration of target compounds, enabling consistent results in enzymatic reactions [41]. For *R. adamsii* leaves of three ages and at different loadings (0.5–2.0 g/100 mL), extracts were obtained with total flavonoid concentrations ranging from 16.58 mg/g in the litterfall leaves sample at a loading of 0.5 g/100 mL to 135.87 mg/g in the first-year leaves sample at a loading of 2.0 g/100 mL (Table 4 and Table S5).

The contents of the main flavonoids in the dry extracts were as follows: 3.41–30.92 mg/g for myricetin 3′-*O*-xylopyranoside, 1.14–24.39 mg/g for quercetin 3-*O*-galactoside, 0–15.22 mg/g for adamsoside IV, 0–14.22 mg/g for quercetin 3-*O*-xylopyranoside, and 1.43–10.67 mg/g for 5-*O*-methylmyricetin 3-*O*-galactoside.

The anti-hPL activities of nine *R. adamsii* extracts yielded IC_50_ values of 30.65–123.63 μg/mL. Notably, two extracts (1Y-2.0: 30.65 μg/mL; 1Y-1.0: 33.47 μg/mL) exhibited lower IC_50_ values than the reference inhibitor orlistat (35.28 μg/mL) under the same experimental conditions (Table 5). However, this comparison should be interpreted with caution because the extracts are complex mixtures containing various compounds, whereas orlistat is a single purified active agent. The apparently lower IC_50_ of an extract does not necessarily indicate greater efficacy in a biological system because synergistic or matrix effects may influence the activity of the mixture. The efficacy of green (IC_50_ 45.67 μg/mL) and black tea extracts (IC_50_ 76.11 μg/mL) was lower than that of orlistat under these conditions and was similar to those of rhododendron extracts 2Y-2.0 (IC_50_ 58.29 μg/mL) and LF-2.0 or 2Y-0.5 (77.05 or 79.24 μg/mL), respectively. These data show that rhododendron extracts with high flavonoid content exhibit potent hPL inhibitory activity, with values comparable to or higher than those of traditional tea beverages under the tested conditions [42,43].

Pearson correlation analysis (*n* = 9 extracts) revealed a significant inverse correlation between total flavonoid content and hPL inhibitory potential (Pearson r = −0.918, R^2^ = 0.8421, *p* ~4.8 × 10^−4^) (Figure 4, Table S6). This strong correlation supports the role of flavonoids in the observed biological effect. However, it does not establish that only flavonoids are responsible for the activity. The potential contribution of other phytochemicals cannot be excluded and requires further experimental confirmation.

To better understand which compounds in the extracts have the greatest influence on the overall effect, 20 flavonoids were examined (Table 5). The highest inhibition rates and lowest IC_50_ values were observed for myricetin (IC_50_ 8.38 μg/mL) and quercetin (IC_50_ 12.34 μg/mL) present in *R. adamsii* leaves, as well as for gossypetin (IC_50_ 8.02 μg/mL), which was present as glycosides. Notably, the attachment of a carbohydrate fragment at any position was associated with reduced inhibitory activity of the tested compounds (IC_50_ value increased), as observed for 3-*O*-glycosides (myricetin 3-*O*-galactoside IC_50_ 45.63 μg/mL; gossypetin glycosides IC_50_ 30.84–31.12 μg/mL; quercetin glycosides IC_50_ 71.08–75.19 μg/mL), and for 3′-*O*-glycosides (myricetin 3′-*O*-glycosides IC_50_ 60.07–62.08 μg/mL). This trend is consistent with the previously described effect of glycosylation, which has been proposed to interfere with molecular interactions, possibly by shielding active centers [33]. However, this mechanistic interpretation was not directly tested in our study. The effect of acylation was reflected both in increased activity, as observed for myricetin 3-*O*-(6″-*O*-benzoyl)-galactoside (IC_50_ 35.22 μg/mL vs. myricetin 3-*O*-galactoside IC_50_ 45.63 μg/mL), and in decreased activity for quercetin 3-*O*-(6″-*O*-acetyl)-galactoside (IC_50_ 79.52 μg/mL vs. quercetin 3-*O*-galactoside IC_50_ 72.65 μg/mL). Dihydroflavonols were less effective lipase inhibitors than flavonols, with IC_50_ values of 105.10 μg/mL for taxifolin, whereas aromadendrin and its glycosides had IC_50_ values > 200 μg/mL. Among the tested compounds, five flavonoids detected in *R. adamsii* leaves showed IC_50_ values comparable to or lower than that of orlistat, including myricetin, quercetin, myricetin 3-*O*-(6″-*O*-benzoyl)-galactoside, gossypetin 3-*O*-arabinopyranoside, and gossypetin 3-*O*-galactoside, suggesting that they may be potential natural lipase inhibitors.

Based on the concentrations of these flavonoids in the extracts and their inhibitory activities as isolated compounds, we tentatively identified the following compounds as candidate contributors to the overall antilipase effect: myricetin, myricetin 3′-*O*-xylopyranoside, and quercetin 3-*O*-galactoside in first-year leaves; myricetin, quercetin 3-*O*-xylopyranoside, and quercetin 3-*O*-galactoside in second-year leaves; and myricetin and quercetin in litterfall leaves (Table S7). However, these calculations assume additive effects and do not account for potential synergistic or antagonistic interactions between compounds in the complex extract. Therefore, this estimate should be considered as a hypothesis for testing in future studies, rather than a definitive conclusion about the actual contributors to the observed activity.

### 3.5. Toxicity Assessment of Sagan Dali Tea Decoction Using the Brine Shrimp Lethality Assay

Although flavonoids are not considered toxic compounds and are classified as safe plant metabolites [44], the potentially negative effects of water decoctions of *R. adamsii* leaves (sagan dali tea) warrant investigation because they have not been studied previously. Analysis of BSL following the application of water decoctions from first-year, second-year, and litterfall *R. adamsii* leaves at a loading of 2.0 g/100 mL across four concentration levels (1, 10, 100, and 1000 μg/mL) showed that, at the maximum concentration, mortality rates of 10%, 7%, and 0% were observed. Subsequent probit analysis showed that even at the highest tested concentration (1000 μg/mL), the mortality rate did not exceed 10% (Table 6 and Table S8).

Because a 50% mortality rate was not observed in the tested concentration range, the LC_50_ value could not be determined accurately from the direct experimental data. Extrapolation of the probit regression curves indicated that the LC_50_ values of *R. adamsii* extracts exceeded 1000 µg/mL. Therefore, based on the empirical data obtained under the tested conditions, we report the LC_50_ as > 1000 μg/mL.

According to Meyer’s toxicity index [45], the LC_50_ values indicate that the sagan dali decoctions exhibit low brine shrimp lethality because their values exceeded the 1000 μg/mL threshold. The toxic reference standard, cyclophosphamide, exhibited an LC_50_ value of 12.5 μg/mL, consistent with active alkylating agents widely used in oncology practice [46]. For comparison, the toxicity of the traditional tea beverages (green and black tea, prepared using a similar technology) was assessed. The LC_50_ values for these teas were 755 and >1000 μg/mL, respectively; thus, they were classified as slightly toxic and non-toxic, respectively. The greater toxicity of green tea compared with sagan dali may be attributable to the alkaloid caffeine, which is absent in *R. adamsii*; its presence leads to greater lethality in brine shrimp [47]. The direct relationship between alkaloid levels in herbal teas and increased brine shrimp toxicity has been discussed previously using American plants as examples [48]. However, it should be emphasized that the BSL assay provides only an initial assessment of acute lethality in a non-mammalian model and does not directly reflect human health implications.

Previous studies have shown that, for the same plant, toxicity can vary with the solvent used, increasing when alcohols more lipophilic than water are used [49]. Extracts of first-year, second-year, and litterfall leaves of *R. adamsii* prepared with 40%, 70%, and 100% methanol had LC_50_ values greater than 1000 μg/mL in the BSL assay, indicating low brine shrimp lethality under the tested conditions. This is consistent with numerous studies on the toxicity of herbal teas that do not contain toxic or highly active compounds (e.g., alkaloids and triterpenes), including sagan dali tea, in which LC_50_ values have been shown to exceed 1000 μg/mL, indicating no acute lethality in the BSL assay for these plant-derived beverages in this biological model [50,51,52].

## 4. Discussion

The age-dependent development of plant organs is associated with various morphological, genetic, and chemical processes that determine the growth and development of an individual organism [12]. The general pattern of chemical changes in plant leaves involves the gradual accumulation of specific metabolites from the young to the adult stage, followed by the retention or consumption of these compounds depending on the organism’s needs [13]. The development of species of the genus *Rhododendron* is no exception and is accompanied by various seasonal and age-dependent changes, including metabolic shifts [4]. A study of the phenolic compound content in the leaves of *R. tomentosum* showed that the greatest diversity of phenolics occurred during the autumn–winter dormancy period, whereas the fruiting period exhibited the lowest diversity. During dormancy, procyanidins (a flavonoid subclass) and phenolic acids predominated, whereas other flavonoids, particularly quercitrin, hyperoside, and isoquercitrin, accumulated during flowering [53]. Statistically significant differences were recorded for the Himalayan species *R. anthopogon*, with variability in nitrogen content, the carbon–nitrogen ratio, total chlorophyll, and proline content observed in leaves of different ages [54]. A decreasing trend in low-molecular-weight organic acids was observed during the transition from fresh foliage to fallen leaves in *R. delavayi*, *R. agastum*, and *R. irroratum*, with oxalic acid as the predominant acid, followed by malic and tartaric acids [55]. The evergreen species *R. catawbiense* responded to seasonal changes through fluctuations in photosynthetic rates, resulting in carotenoid accumulation during summer [56]. In *R. aganniphum*, *R. nyingchiense*, *R. wardii*, and *R. triflorum*, the highest levels of proline, chlorophylls, and carotenoids were observed in winter, while the total content of soluble sugars increased in spring and autumn [57].

Adams’ rhododendron is a characteristic representative of evergreen rhododendrons, biochemically characterized by its ability to accumulate various groups of phenolic compounds, especially flavonoids, which occur in the plant as aglycones and glycosides from the quercetin, myricetin, taxifolin, aromadendrin, and other groups [4,5,19,20]. The two-year life cycle of *R. adamsii* leaves suggests changes in flavonoid levels, the main phenolic components. Studies of *R. adamsii* leaves of different ages over three years (2021–2023) revealed these shifts, with the greatest diversity and concentration of flavonoids and other phenols found in first-year leaves, followed by a decrease in the number of metabolites in second-year leaves and the disappearance of some compounds in litterfall leaves. Flavonoid glycosides present in first-year leaves at levels of 3833–3992 mg/100 g decreased to 2331–2614 mg/100 g in second-year leaves and further to 489–580 mg/100 g in litterfall leaves. A possible explanation for this phenomenon is the gradual increase in the rate of hydrolytic processes [58], which may account for the increase in aglycone levels from 220–248 mg/100 g in first-year leaves to 527–568 mg/100 g in litterfall leaves. However, other processes, including leaching, oxidation, microbial activity, and post-abscission degradation, may also contribute to the observed changes in litterfall leaves.

A distinctive feature of *R. adamsii* as a food species is the use of leaves of different ages to prepare a decoction, or sagan dali tea. The data obtained indicate, for the first time, that “different-aged” sagan dali tea blends may differ in flavonoid content and composition, with young teas characterized as high-flavonoid blends and old teas as low-flavonoid blends. A similar trend had previously been observed in teas of different ages from *Camellia sinensis*, for which “aging” is not a criterion for reducing the quality of the final infusion, but rather simply broadens the range of tea beverages [59].

The transfer of flavonoids from the plant matrix into the aqueous phase during boiling is an important step in producing sagan dali tea because it affects the tea’s chemical properties [60]. At loadings of 0.5–2.0 g/100 mL, decoctions prepared from first-year leaves contain 15–63 mg/100 mL of flavonoids, those prepared from second-year leaves contain 8–35 mg/100 mL, and those prepared from leaf litter contain 1–7 mg/100 mL. This flavonoid content places sagan dali close to the levels found in green tea decoctions (30–60 mg/100 mL), some ready-to-drink green tea beverages (10–40 mg/100 mL) [61], and loose-leaf teas (39–57 mg/100 mL) [62]. The global trend toward diversifying food products, including functional products such as herbal teas with sagan dali, supports recommending broader use of *R. adamsii* leaves as a flavonoid source.

Early ethnopharmacological data on the use of *R. adamsii* to treat disorders of lipid metabolism support considering extracts, including sagan dali tea, as lipase inhibitors, which represent the initial stage of therapy in most cases of lipid disorders. The diversity of flavonoids found in *R. adamsii* suggests an antilipase effect, as previously demonstrated for this group of natural phenolics [27]. Quercetin, which is present in sagan dali tea in glycosidic form, is a well-known inhibitor of pancreatic lipase that binds to the enzyme’s noncompetitive domain; it also reduces postprandial serum triglyceride levels and increases fecal fat excretion [63]. Myricetin, also found in *R. adamsii*, forms aggregates in the intestines and inhibits lipase through a sequestering mechanism [64]. In our study, we demonstrated for the first time the anti-lipase activity of *R. adamsii* leaf decoctions, which correlated well with the flavonoid content of the extract. The IC_50_ values were comparable to or lower than those of orlistat in high-flavonoid samples obtained from first-year leaves under the same experimental conditions. However, this comparison should be interpreted cautiously, because extracts are complex mixtures, whereas orlistat is a pure compound. Moreover, these findings do not demonstrate anti-obesity activity, triglyceride reduction, or therapeutic efficacy in vivo because in vitro enzyme inhibition does not necessarily translate into clinical effects. A similar trend linking the high polyphenol content of tea beverages to their high antilipase activity was observed in green [65] and black tea extracts [66], which were active not only in in vitro studies [67], but also lowered serum triglyceride levels and reduced LDL cholesterol levels in high-fat-fed animals [68].

Despite the extensive information available on plant lipase inhibitors [26], most experiments have focused on porcine pancreatic lipase, whereas data on the effects of metabolites on hPL remain limited. Recently, several *R. adamsii* flavonoids, including quercetin, myricetin, quercetin 3-*O*-xylopyranoside [69], and quercetin 3-*O*-glucoside [70], have been evaluated as hPL inhibitors, while the majority of flavonoids in *R. adamsii* leaves remain understudied in relation to hPL. Our data revealed, for the first time, that the 3-*O*-galactoside and 3-*O*-(6″-*O*-benzoyl)-galactoside of myricetin, the 3-*O*-arabinopyranoside and 3-*O*-galactoside of gossypetin, and 5-*O*-methylmyricetin 3-*O*-galactoside exhibited greater or comparable inhibitory efficacy in the hPL-based assay. These compounds are found not only in *R. adamsii* but also in other rhododendrons [1,2,3] and Ericaceae species [71]. Their detection in plants may serve as a criterion indicating the likely presence of antilipase activity in extracts. The inhibitory effect was moderate for quercetin 3-*O*-glycosides, found in most plant metabolomes [72], and myricetin 3′-*O*-glycosides, associated with rare phenoloids [73].

The widespread use of *R. adamsii* as a tea plant has been overshadowed by early reports of grayanotoxin [30], a highly toxic component that was not detected in any subsequent studies performed with more sensitive equipment than that used in the original report [4,5,11,19,20]. Preliminary toxicity studies of *R. adamsii* in mice yielded an LD_50_ value > 3000 mg/kg [74], enabling the tincture to be classified as having low toxicity [45]. Data on the toxicity of *R. adamsii* water decoctions or sagan dali tea could not be found. Thus, in the brine shrimp assay, decoctions of *R. adamsii* of various ages exhibited low brine shrimp lethality (LC_50_ > 1000 µg/mL), similar to that of black tea (LC_50_ > 1000 µg/mL) and lower than that of green tea (LC_50_ = 755 µg/mL) under the same conditions [75]. Other rhododendron species exhibit varying levels of toxicity depending on the presence or absence of grayanotoxins. In non-food species, grayanotoxin concentrations range from 0.05% in *R. mucronatum* and *R. galactinum* [76] to 5% in *R. luteum* and *R. ponticum* [77]. In contrast, the tea plants *R. ferrugineum*, *R. cuneatum*, and *R. hirsutum* tested negative for grayanotoxins [76] and exhibited low brine shrimp lethality under the tested conditions, with LC_50_ values > 1000 μg/mL, as also observed for *R. redowskianum*, *R. lapponicum* [78], and *R. macrophyllum* [79]. Although these results provide only an exploratory characterization of sagan dali tea, they support further in-depth research on this beverage, including targeted quantification of grayanotoxins, to obtain conclusive toxicological data.

It should be emphasized that the phenolic profile and content of sagan dali tea are closely associated with the age of *R. adamsii* leaves. Our findings indicate a strong correlation between the inhibitory effect of sagan dali tea on hPL and flavonoid levels in leaves of different ages. First-year leaves consistently showed higher flavonoid content and greater bioactivity than litterfall. The antilipase components, including myricetin, quercetin, and gossypetin, along with their glycosides, appeared to contribute to the observed biological effect because they were present at high concentrations. However, a direct contribution cannot be established from the present data. According to the brine shrimp bioassay, sagan dali tea showed no acute lethality in the BSL assay regardless of leaf age, providing preliminary evidence of low brine shrimp lethality under the tested conditions. However, this does not establish its safety for human consumption. Our results offer valuable insights for *R. adamsii* harvesters and sagan dali processors, helping guide the selection of the optimal leaf age to maximize bioactivity while minimizing impacts on wild populations.

## 5. Conclusions

This study presents the first comprehensive evaluation of water decoctions (sagan dali tea) from *Rhododendron adamsii* leaves of different ages, revealing that leaf age significantly affects both flavonoid content and biological activity. First-year leaves showed the highest flavonoid levels (3833–3992 mg/100 g) and the greatest hPL inhibitory potency (IC_50_ 30.65–33.47 μg/mL), which is comparable to orlistat. A strong correlation (R^2^ = 0.8421, *p* < 0.05) supports the key role of flavonoids in this activity, with myricetin, quercetin, gossypetin, and their glycosides identified as the primary candidate active principles. However, the in vitro nature of the assay, preliminary toxicity data, and unknown synergistic effects limit these findings. To confirm the efficacy and safety of sagan dali tea, further research is required using in vivo rodent models, as well as bioassay-guided fractionation to identify the full spectrum of active principles.

## Figures and Tables

**Figure 1:**
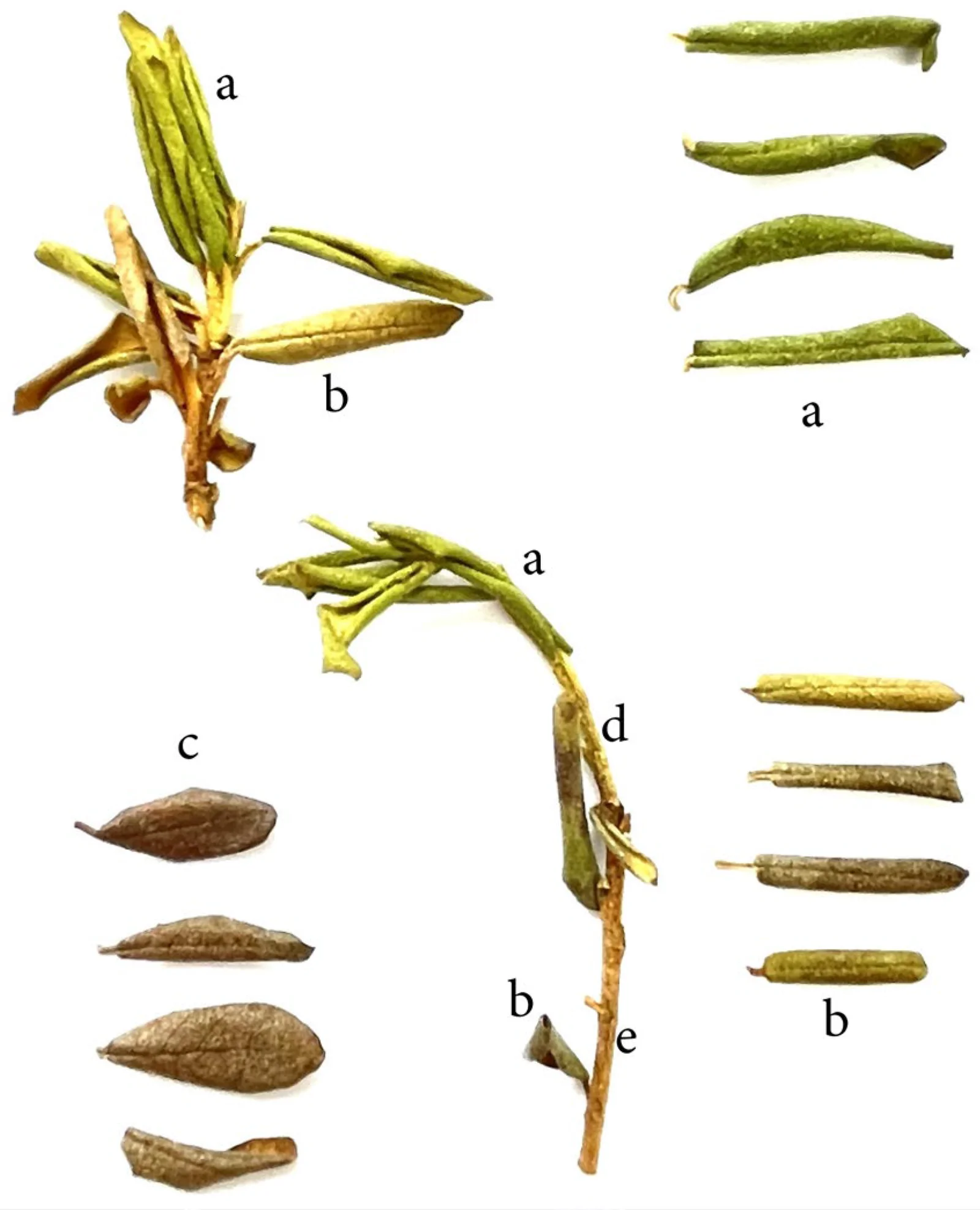
Leaves of *Rhododendron adamsii* Rehder at various ages: first-year (**a**), second-year (**b**), and litterfall (**c**), together with first-year (**d**) and second-year (**e**) shoots.

**Figure 2:**
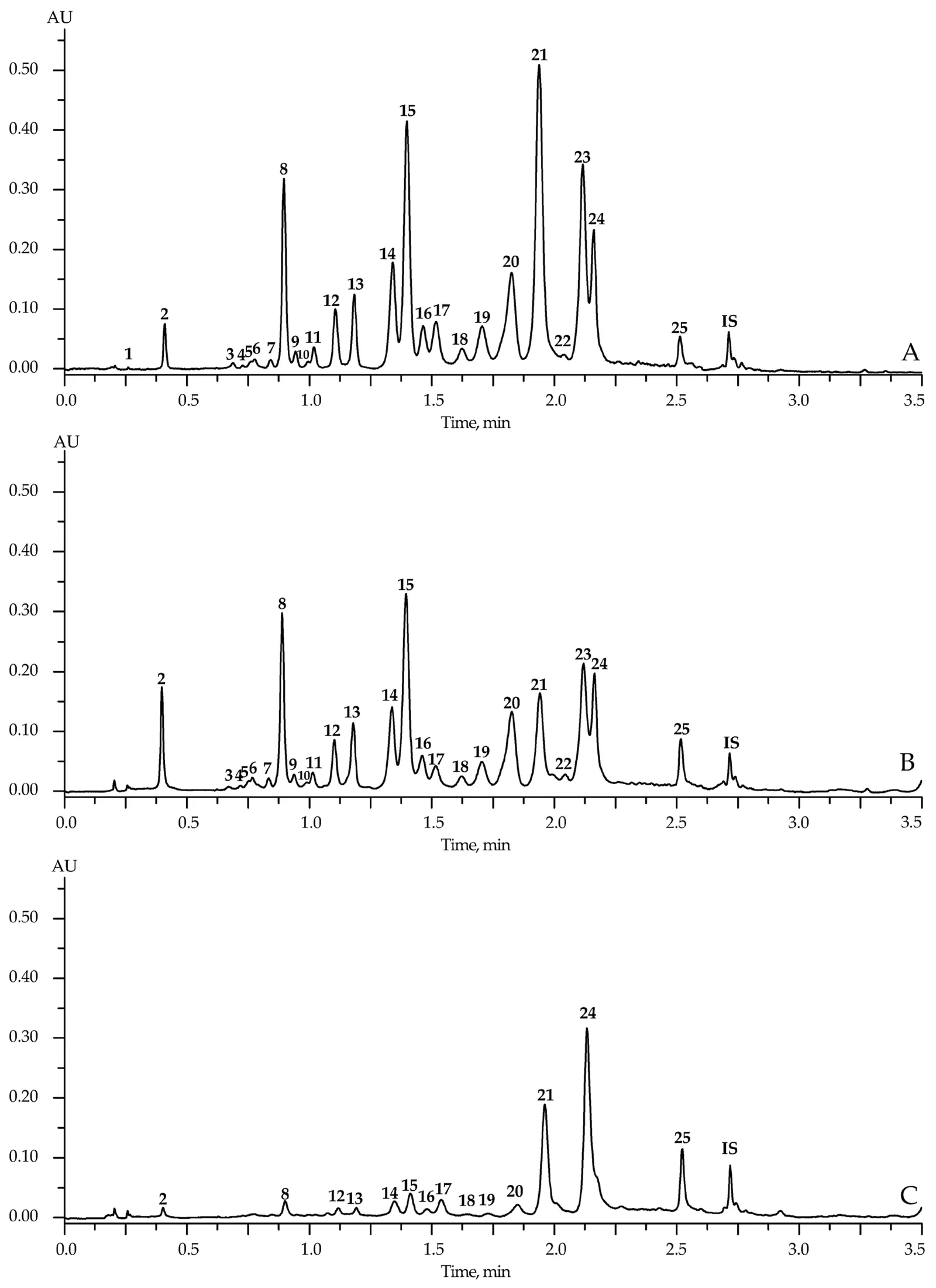
Fast MC-HPLC chromatograms of methanolic extracts from first-year (**A**), second-year (**B**), and litterfall (**C**) *R. adamsii* leaves (all samples were collected in 2021), detected by PDA at 270 nm. The identities of compounds **1**–**25** are shown in Table 1. IS—internal standard (isorhamnetin).

**Figure 3:**
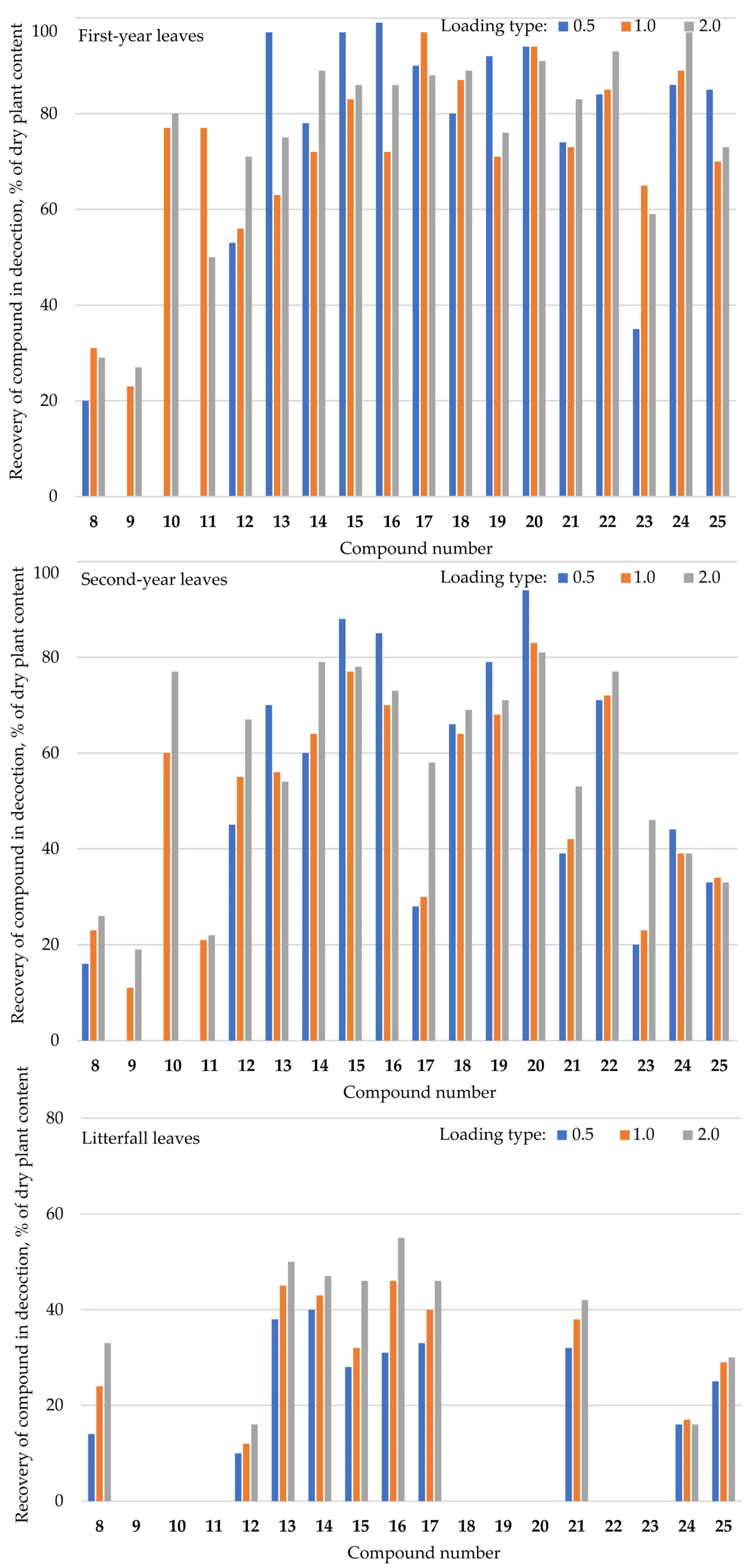
Recovery of flavonoids in decoctions prepared from first-year, second-year, and litterfall of *R. adamsii* leaves. Compounds are numbered as shown in Table 1.

**Figure 4:**
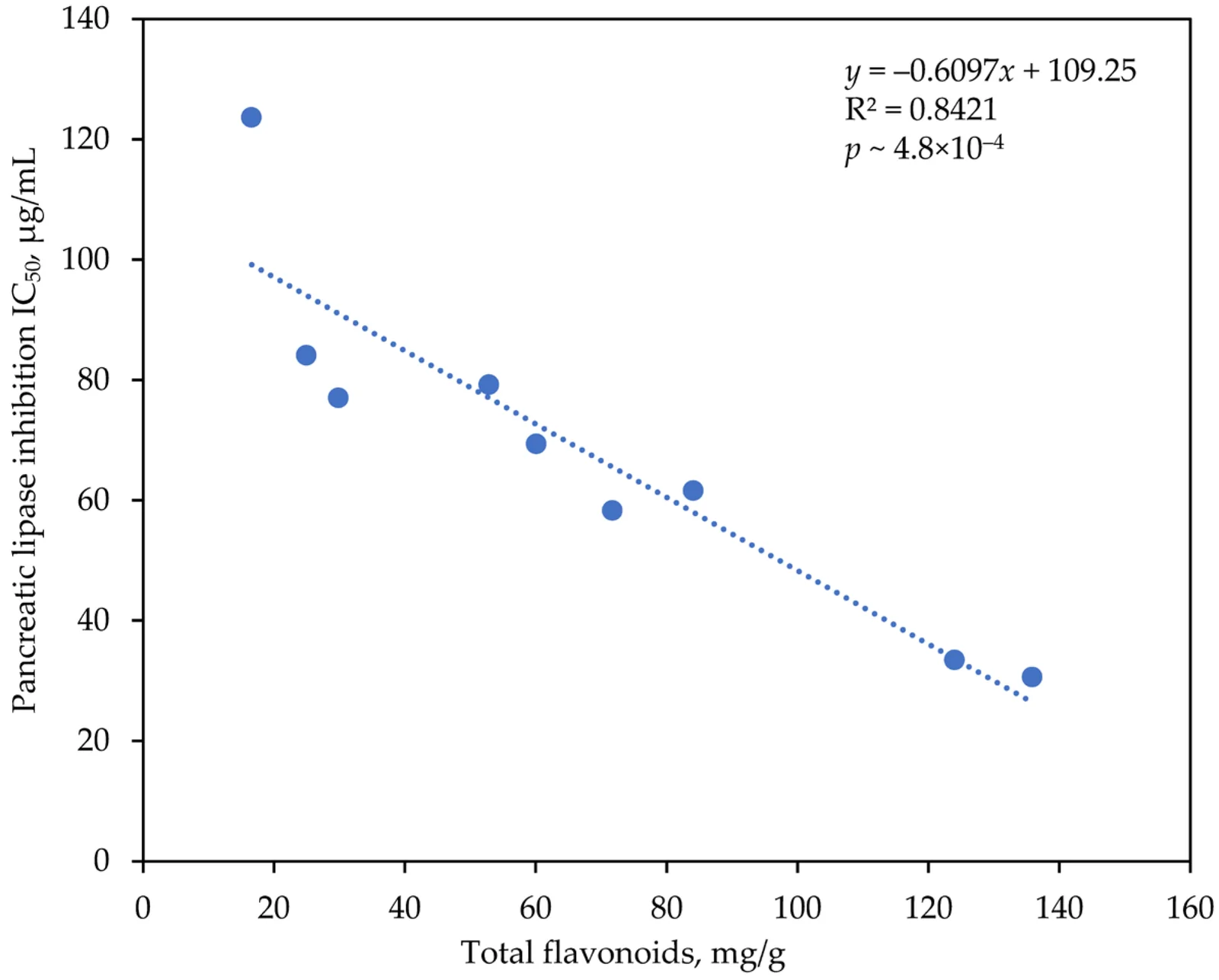
Pearson correlation scatter plot showing the relationship between total flavonoid content (mg/g, HPLC) and human pancreatic lipase inhibitory potential (IC_50_, μg/mL) of *R. adamsii* leaf extracts, including the regression equation, coefficient of determination (*R*^2^), and *p*-value.

**Table 1:** Retention times (t_R_), molecular formulas (MF), deprotonated ions ([M − H]^−^), identification levels (IL [37]), and occurrence of compounds **1**–**25** detected in first-year (1Y), second-year (2Y), and litterfall (LF) of *R. adamsii* leaves.

No	T_R_, min	Compound	MF	[M − H]^−^, *m*/*z*	IL	Found in *		
						1Y	2Y	LF
**1**	0.26	Vanillic acid di-*O*-hexoside	C_20_H_28_O_14_	491	2	+	−	−
**2**	0.40	Vanillic acid 4-*O*-glucoside	C_14_H_18_O_9_	329	1	+	+	+
**3**	0.71	Vanillic acid *O*-hexoside	C_14_H_18_O_9_	329	2	+	+	−
**4**	0.72	Taxifolin 3-*O*-glucoside (glucodistylin)	C_21_H_22_O_12_	465	1	+	+	−
**5**	0.75	Vanilloyl quinic acid	C_15_H_18_O_9_	341	2	+	+	−
**6**	0.77	5-*O*-Caffeoyl-quinic acid, *trans*-isomer	C_16_H_18_O_9_	353	1	+	+	−
**7**	0.80	Caffeic acid	C_9_H_8_O_4_	179	1	+	+	−
**8**	0.89	Gossypetin 3-*O*-galactoside	C_21_H_20_O_13_	479	1	+	+	+
**9**	0.93	Myricetin 3-*O*-galactoside (gmelinoside I)	C_21_H_20_O_13_	479	1	+	+	−
**10**	0.98	Gossypetin *O*-pentoside	C_20_H_18_O_12_	449	2	+	+	−
**11**	1.00	Gossypetin 3-*O*-arabinopyranoside (rhododeside A)	C_20_H_18_O_12_	449	1	+	+	−
**12**	1.09	Aromadendrin 3-*O*-glucoside	C_21_H_22_O_11_	449	1	+	+	+
**13**	1.18	Taxifolin 3-*O*-arabinopyranoside	C_20_H_20_O_11_	435	1	+	+	+
**14**	1.32	5-*O*-Methylmyricetin 3-*O*-galactoside	C_22_H_22_O_13_	493	1	+	+	+
**15**	1.38	Quercetin 3-*O*-galactoside (hyperoside)	C_21_H_20_O_12_	463	1	+	+	+
**16**	1.46	Quercetin 3-*O*-glucoside (isoquercitrin)	C_21_H_20_O_12_	463	1	+	+	+
**17**	1.50	Myricetin 3′-*O*-glucoside (cannabiscitrin)	C_21_H_20_O_13_	479	1	+	+	+
**18**	1.61	Quercetin 3-*O*-arabinofuranoside (avicularin)	C_20_H_18_O_11_	433	1	+	+	+
**19**	1.70	Quercetin 3-*O*-arabinopyranoside (guaijaverin)	C_20_H_18_O_11_	433	1	+	+	+
**20**	1.79	Quercetin 3-*O*-xylopyranoside (reynoutrin)	C_20_H_18_O_11_	433	1	+	+	+
**21**	1.92	Myricetin 3′-*O*-xylopyranoside	C_20_H_18_O_12_	449	1	+	+	+
**22**	2.01	Quercetin 3-*O*-(6″-*O*-acetyl)-galactoside	C_23_H_22_O_13_	505	1	+	+	−
**23**	2.07	Myricetin 3-*O*-(6″-*O*-benzoyl)-galactoside (adamsoside IV)	C_28_H_24_O_14_	583	1	+	+	−
**24**	2.14	Myricetin	C_15_H_10_O_8_	317	1	+	+	+
**25**	2.47	Quercetin	C_15_H_10_O_7_	301	1	+	+	+
	2.67	Internal standard (isorhamnetin)	C_16_H_12_O_7_	315	1			

* Occurrence of compounds: “+”―present, “−”―absent.

**Table 2:** Content (mg/100 g) of compounds **1**–**25** in first-year (1Y), second-year (2Y), and litterfall (LF) of *R. adamsii* leaves in samples from 2021–2023.

No	Compound	2021			2022			2023		
		1Y	2Y	LF	1Y	2Y	LF	1Y	2Y	LF
**1**	Vanillic acid di-*O*-hexoside	2	0	0	1	0	0	2	0	0
**2**	Vanillic acid 4-*O*-glucoside	30	57	1	20	48	1	41	42	0
**3**	Vanillic acid *O*-hexoside	7	2	0	9	2	0	11	2	0
**4**	Taxifolin 3-*O*-glucoside (glucodistylin)	9	4	0	12	5	0	9	3	0
**5**	Vanilloyl quinic acid	11	9	0	15	8	0	11	7	0
**6**	5-*O*-Caffeoyl-quinic acid, *trans*-isomer	14	13	0	9	11	0	22	10	0
**7**	Caffeic acid	11	8	0	17	6	0	28	6	0
**8**	Gossypetin 3-*O*-galactoside	306	306	30	327	304	22	311	295	24
**9**	Myricetin 3-*O*-galactoside (gmelinoside I)	30	27	0	25	22	0	20	17	0
**10**	Gossypetin *O*-pentoside	4	2	0	2	1	0	3	1	0
**11**	Gossypetin 3-*O*-arabinopyranoside (rhododeside A)	31	27	0	45	31	0	34	26	0
**12**	Aromadendrin 3-*O*-glucoside	128	111	33	135	91	34	115	92	38
**13**	Taxifolin 3-*O*-arabinopyranoside	156	149	27	176	132	22	190	128	18
**14**	5-*O*-Methylmyricetin 3-*O*-galactoside	280	211	54	292	203	49	291	199	40
**15**	Quercetin 3-*O*-galactoside (hyperoside)	672	564	65	698	536	67	714	510	60
**16**	Quercetin 3-*O*-glucoside (isoquercitrin)	122	97	20	123	103	16	138	92	10
**17**	Myricetin 3′-*O*-glucoside (cannabiscitrin)	117	63	51	129	60	42	111	59	42
**18**	Quercetin 3-*O*-arabinofuranoside (avicularin)	45	32	6	32	23	1	37	21	2
**19**	Quercetin 3-*O*-arabinopyranoside (guaijaverin)	137	83	8	128	76	10	141	74	7
**20**	Quercetin 3-*O*-xylopyranoside (reynoutrin)	361	309	0	383	279	0	358	269	0
**21**	Myricetin 3′-*O*-xylopyranoside	881	276	286	917	244	275	915	250	248
**22**	Quercetin 3-*O*-(6″-*O*-acetyl)-galactoside	14	7	0	12	4	0	10	4	0
**23**	Myricetin 3-*O*-(6″-*O*-benzoyl)-galactoside (adamsoside IV)	540	346	0	556	317	0	521	291	0
**24**	Myricetin	208	285	427	195	302	451	186	293	448
**25**	Quercetin	40	64	100	32	82	117	34	82	106
	Total vanillates (Va)	50	68	1	45	58	1	65	51	0
	Total caffeates (Ca)	25	21	0	26	17	0	50	16	0
	Total non-flavonoids (Σ_Va, Ca_)	75	89	1	71	75	1	115	67	0
	Total taxifolins (Tx)	165	153	27	188	137	22	199	131	18
	Total aromadendrins (Ar)	128	111	33	135	91	34	115	92	38
	Total dihydroflavonols (Σ_Tx, Ar_)	293	264	60	323	228	56	314	223	56
	Total gossypetins (Gs)	341	335	30	374	336	22	348	322	24
	Total myricetins (My)	1776	997	764	1822	945	768	1753	910	738
	Total 5-*O*-methylmyricetins (5MMy)	280	211	54	292	203	49	291	199	40
	Total quercetins (Qu)	1391	1156	199	1408	1103	211	1432	1052	185
	Total flavonols (Σ_Gs, My, 5MMy, Qu_)	3788	2699	1047	3896	2587	1050	3824	2483	987
	Total flavonoids (Σ_Tx, Ar, Gs, My, 5MMy, Qu_)	4081	2963	1107	4219	2815	1106	4138	2706	1043
	Total flavonoid glycosides	3833	2614	580	3992	2431	538	3918	2331	489
	Total flavonoid aglycones	248	349	527	227	384	568	220	375	554
	Total phenolics (Σ_Va, Ca, Tx, Ar, Gs, My, 5MMy, Qu_)	4156	3052	1108	4290	2890	1107	4253	2773	1043
	Percentage of non-flavonoids to total phenolics	1.8	2.9	0.1	1.7	2.6	0.1	2.7	2.4	0
	Percentage of flavonoids to total phenolics	98.2	97.1	99.9	98.3	97.4	99.9	97.3	97.6	100

Non-flavonoids = vanillic acid and caffeic acid derivatives; Dihydroflavonols = taxifolin and aromadendrin derivatives; flavonols = gossypetin, myricetin, 5-*O*-methylmyricetin, and quercetin derivatives; Flavonoids = flavonols + dihydroflavonols; total phenolics = non-flavonoids + flavonoids.

**Table 3:** Flavonoid content (μg/100 mL) in decoctions of first-year (1Y), second-year (2Y), and litterfall (LF) of *R. adamsii* leaves with loadings of 0.5, 1.0, and 2.0 g/100 mL.

Compound	1Y			2Y			LF		
	0.5	1.0	2.0	0.5	1.0	2.0	0.5	1.0	2.0
Gossypetin 3-*O*-galactoside	308	963	1829	250	704	1604	21	72	198
Myricetin 3-*O*-galactoside (gmelinoside I)	0	70	163	0	32	104	0	0	0
Gossypetin *O*-pentoside	0	31	64	0	12	31	0	0	0
Gossypetin 3-*O*-arabinopyranoside (rhododeside A)	0	240	310	0	57	121	0	0	0
Aromadendrin 3-*O*-glucoside	340	721	1837	255	617	1497	18	41	107
Taxifolin 3-*O*-arabinopyranoside	760	995	2356	525	840	1612	52	124	273
5-*O*-Methylmyricetin 3-*O*-galactoside	1092	2022	4988	637	1371	3374	108	235	509
Quercetin 3-*O*-galactoside (hyperoside)	3279	5596	11,562	2494	4349	8853	93	211	598
Quercetin 3-*O*-glucoside (isoquercitrin)	604	881	2113	414	688	1417	31	93	222
Myricetin 3′-*O*-glucoside (cannabiscitrin)	532	1141	2067	91	191	732	86	209	471
Quercetin 3-*O*-arabinofuranoside (avicularin)	181	395	805	107	207	447	0	0	0
Quercetin 3-*O*-arabinopyranoside (guaijaverin)	633	981	2099	329	570	1188	0	0	0
Quercetin 3-*O*-xylopyranoside (reynoutrin)	1701	3413	6614	1466	2565	5037	0	0	0
Myricetin 3′-*O*-xylopyranoside	3282	6443	14,637	542	1163	2959	463	1108	2432
Quercetin 3-*O*-(6″-*O*-acetyl)-galactoside	59	120	263	25	51	108	0	0	0
Myricetin 3-*O*-(6″-*O*-benzoyl)-galactoside (adamsoside IV)	971	3557	6445	357	802	3233	0	0	0
Myricetin	899	1864	4066	641	1121	2240	354	753	1440
Quercetin	170	282	585	136	221	658	128	296	614
Total taxifolins (Tx)	760	995	2356	525	840	1612	52	124	273
Total aromadendrins (Ar)	340	721	1837	255	617	1497	18	41	107
Total dihydroflavonols (Σ_Tx, Ar_)	1100	1716	4193	780	1457	3109	70	165	380
Total gossypetins (Gs)	308	1234	2203	250	773	1756	21	72	198
Total myricetins (My)	5684	13,075	27,378	1631	3309	9268	903	2070	4343
Total 5-*O*-methylmyricetins (5MMy)	1092	2022	4988	637	1371	3374	108	235	509
Total quercetins (Qu)	6627	11,668	24,041	4941	8651	17,485	252	600	1434
Total flavonols (Σ_Gs, My, 5MMy, Qu_)	13,711	27,999	58,610	7459	14,104	31,883	1284	2977	6484
Total flavonoids (Σ_Tx, Ar, Gs, My, 5MMy, Qu_)	14,811	29,715	62,803	8239	15,561	34,992	1354	3142	6864
Total flavonoid glycosides	13,742	27,569	58,152	7492	14,219	32,317	872	2093	4810
Total flavonoid aglycones	1069	2146	4651	747	1342	2675	482	1049	2054

Dihydroflavonols = taxifolin and aromadendrin derivatives; flavonols = gossypetin, myricetin, 5-*O*-methylmyricetin, and quercetin derivatives; Flavonoids = flavonols + dihydroflavonols.

**Table 4:** Flavonoid content (mg/g) in dry extracts from first-year (1Y), second-year (2Y), and litterfall (LF) of *R. adamsii* leaves with loadings of 0.5, 1.0, and 2.0 g/100 mL.

Compound	1Y			2Y			LF		
	0.5	1.0	2.0	0.5	1.0	2.0	0.5	1.0	2.0
Gossypetin 3-*O*-galactoside	1.73	4.02	3.96	1.62	2.75	3.26	0.35	0.62	0.85
Myricetin 3-*O*-galactoside (gmelinoside I)	0.00	0.41	0.45	0.00	0.12	0.22	0.00	0.00	0.00
Gossypetin *O*-pentoside	0.00	0.14	0.16	0.00	0.08	0.11	0.00	0.00	0.00
Gossypetin 3-*O*-arabinopyranoside (rhododeside A)	0.00	1.10	0.73	0.00	0.35	0.30	0.00	0.00	0.00
Aromadendrin 3-*O*-glucoside	2.36	3.06	4.02	1.73	2.53	3.06	0.26	0.35	0.51
Taxifolin 3-*O*-arabinopyranoside	4.59	4.16	5.21	3.49	3.39	3.29	0.71	0.99	1.49
5-*O*-Methylmyricetin 3-*O*-galactoside	6.83	8.07	10.67	4.04	5.21	6.79	1.43	1.93	2.21
Quercetin 3-*O*-galactoside (hyperoside)	18.96	22.79	24.39	15.73	16.35	17.93	1.14	1.79	2.69
Quercetin 3-*O*-glucoside (isoquercitrin)	3.42	3.67	4.69	2.69	2.76	2.94	0.42	0.70	1.14
Myricetin 3′-*O*-glucoside (cannabiscitrin)	3.11	4.85	4.30	0.64	0.85	1.59	1.01	1.73	2.05
Quercetin 3-*O*-arabinofuranoside (avicularin)	1.09	1.86	1.82	0.73	0.89	0.98	0.00	0.00	0.00
Quercetin 3-*O*-arabinopyranoside (guaijaverin)	3.59	4.21	4.71	2.16	2.23	2.49	0.00	0.00	0.00
Quercetin 3-*O*-xylopyranoside (reynoutrin)	9.63	14.12	14.22	9.23	9.63	10.14	0.00	0.00	0.00
Myricetin 3′-*O*-xylopyranoside	19.37	26.96	30.92	3.41	4.37	5.94	5.49	8.55	10.12
Quercetin 3-*O*-(6″-*O*-acetyl)-galactoside	0.43	0.53	0.63	0.22	0.25	0.26	0.00	0.00	0.00
Myricetin 3-*O*-(6″-*O*-benzoyl)-galactoside (adamsoside IV)	5.63	15.22	14.93	2.35	3.05	6.73	0.00	0.00	0.00
Myricetin	2.31	7.69	8.63	4.11	4.36	4.76	4.18	5.97	6.18
Quercetin	1.03	1.14	1.43	0.73	0.93	0.93	1.59	2.36	2.62
Total taxifolins (Tx)	4.59	4.16	5.21	3.49	3.39	3.29	0.71	0.99	1.49
Total aromadendrins (Ar)	2.36	3.06	4.02	1.73	2.53	3.06	0.26	0.35	0.51
Total dihydroflavonols (Σ_Tx, Ar_)	6.95	7.22	9.23	5.22	5.92	6.35	0.97	1.34	2.00
Total gossypetins (Gs)	1.73	5.26	4.85	1.62	3.18	3.67	0.35	0.62	0.85
Total myricetins (My)	30.42	55.13	59.23	10.51	12.75	19.24	10.68	16.25	18.35
Total 5-*O*-methylmyricetins (5MMy)	6.83	8.07	10.67	4.04	5.21	6.79	1.43	1.93	2.21
Total quercetins (Qu)	38.15	48.32	51.89	31.49	33.04	35.67	3.15	4.85	6.45
Total flavonols (Σ_Gs, My, 5MMy, Qu_)	77.13	116.78	126.64	47.66	54.18	65.37	15.61	23.65	27.86
Total flavonoids (Σ_Tx, Ar, Gs, My, 5MMy, Qu_)	84.08	124.00	135.87	52.88	60.10	71.72	16.58	24.99	29.86
Total flavonoid glycosides	80.74	115.17	125.81	48.04	54.81	66.03	10.81	16.66	21.06
Total flavonoid aglycones	3.34	8.83	10.06	4.84	5.29	5.69	5.77	8.33	8.80

Dihydroflavonols = taxifolin and aromadendrin derivatives; flavonols = gossypetin, myricetin, 5-*O*-methylmyricetin, and quercetin derivatives; Flavonoids = flavonols + dihydroflavonols.

**Table 5:** Human pancreatic lipase inhibition by decoction extracts from first-year (1Y), second-year (2Y), and litterfall (LF) of *R. adamsii* leaves at loadings of 0.5, 1.0, and 2.0 g/100 mL, and by selected compounds.

Extracts	IC_50_, μg/mL	Compounds	IC_50_, μg/mL
1Y-0.5	61.59 ± 1.23 ^e^	Myricetin	8.38 ± 0.15 ^a^
1Y-1.0	33.47 ± 0.61 ^b^	Myricetin 3-*O*-galactoside	45.63 ± 0.86 ^e^
1Y-2.0	30.65 ± 0.59 ^a^	Myricetin 3-*O*-(6″-*O*-benzoyl)-galactoside	35.22 ± 0.72 ^d^
2Y-0.5	79.24 ± 1.63 ^g^	Myricetin 3′-*O*-glucoside	62.08 ± 0.99 ^f^
2Y-1.0	69.39 ± 1.45 ^f^	Myricetin 3′-*O*-xylopyranoside	60.07 ± 1.29 ^f^
2Y-2.0	58.29 ± 1.28 ^d^	5-*O*-Methylmyricetin 3-*O*-galactoside	49.35 ± 1.14 ^e^
LF-0.5	123.63 ± 2.63 ^i^	Quercetin	12.34 ± 0.25 ^b^
LF-1.0	84.12 ± 1.65 ^h^	Quercetin 3-*O*-arabinofuranoside	71.18 ± 1.49 ^g^
LF-2.0	77.05 ± 1.50 ^g^	Quercetin 3-*O*-arabinopyranoside	72.95 ± 1.45 ^g^
Green tea	45.67 ± 0.92 ^c^	Quercetin 3-*O*-xylopyranoside	71.08 ± 1.12 ^g^
Black tea	76.11 ± 1.56 ^g^	Quercetin 3-*O*-glucoside	75.19 ± 1.58 ^h^
		Quercetin 3-*O*-galactoside	72.65 ± 1.45 ^g^
		Quercetin 3-*O*-(6″-*O*-acetyl)-galactoside	79.52 ± 1.52 ^i^
		Gossypetin	8.02 ± 0.14 ^a^
		Gossypetin 3-*O*-arabinopyranoside	30.84 ± 0.63 ^c^
		Gossypetin 3-*O*-galactoside	31.12 ± 0.70 ^c^
		Aromadendrin	>200
		Aromadendrin 3-*O*-glucoside	>200
		Taxifolin	105.10 ± 4.42 ^i^
		Taxifolin 3-*O*-arabinopyranoside	>200
		Orlistat (reference)	35.28 ± 0.77 ^d^

Values with different letters (a–i) indicate statistically significant differences among groups at *p* < 0.05 according to one-way ANOVA.

**Table 6:** Values of LC_50_ (μg/mL) for *R. adamsii* extracts and reference samples in the brine shrimp assay.

Sample	Solvent Type (Toxicity Level *)			
	0% MeOH	40% MeOH	70% MeOH	100% MeOH
First-year leaves	>1000 (NT)	>1000 (NT)	>1000 (NT)	>1000 (NT)
Second-year leaves	>1000 (NT)	>1000 (NT)	>1000 (NT)	>1000 (NT)
Litterfall leaves	>1000 (NT)	>1000 (NT)	>1000 (NT)	>1000 (NT)
Green tea	755 (WT)			
Black tea	>1000 (NT)			
Cyclophosphamide	12.5 (T)			

* According to Meyer’s toxicity index [45]: LC_50_ < 100 µg/mL―toxic (T); 100 µg/mL < LC_50_ < 500 µg/mL―moderately toxic; 500 µg/mL < LC_50_ < 1000 µg/mL―weakly toxic (WT); LC_50_ > 1000 µg/mL―non-toxic (NT).

## Data Availability

The original contributions presented in this study are included in the article and Supplementary Materials. Further inquiries may be directed to the corresponding author.

## Supplementary Materials

The following supporting information can be downloaded at: https://www.mdpi.com/article/10.3390/foods15193433/s1, Table S1; *Rhododendron adamsii* sample description; Table S2: Regression equations, correlation coefficients (r^2^), standard deviation (S_YX_), limits of detection (LOD), limits of quantification (LOQ) and linear ranges for reference standards; Table S3: Content (mg/100 g) of compounds **1**–**25** in first-year (1Y), second-year (2Y), and litterfall (LF) leaves of *R. adamsii* (raw data); Table S4: Recovery (% of inital content) of selected compounds in decoctions of first-year (1Y), second-year (2Y), and litterfall (LF) leaves of *R. adamsii* with 0.5, 1.0, and 2.0 g/100 mL loading (raw data); Table S5: Content (mg/g) of flavonoids and extractives yields in dry extracts of decoctions of first-year (1Y), second-year (2Y), and litterfall (LF) leaves of *R. adamsii* with 0.5, 1.0, and 2.0 g/100 mL loading; Table S6: Correlation coefficients (R^2^) and p-values for lineal correlations “extract pancreatic lipase inhibition, IC_50_, μg/mL – compound content in extract, mg/g”; Table S7: Calculated impact of individual flavonoids on the overall bioeffect (human pancreatic lipase inhibitory potential) of dry extracts of first-year (1Y), second-year (2Y), and litterfall (LF) leaves of *R. adamsii* with 0.5, 1.0, and 2.0 g/100 mL loading; Table S8: Raw data from the brine shrimp lethality assay (probit regression analysis).

## Abbreviations

The following abbreviations are used throughout this manuscript:

BSL	Brine shrimp lethality
hPL	Human pancreatic lipase
HPLC-PDA-Q-TOF-MS	High-performance chromatography with photodiode-array and quadrupole time-of-flight mass spectrometric detection
IC_50_	Half-maximal inhibitory concentration
LC-MS	Liquid chromatography–mass spectrometry
LC_50_	Median lethal concentration

## References

[1] Qiang Y., Zhou B., Gao K. (2011). Chemical constituents of plants from the genus *Rhododendron*. Chem. Biodivers..

[2] Popescu R., Kopp B. (2013). The genus *Rhododendron*: An ethnopharmacological and toxicological review. J. Ethnopharmacol..

[3] Han M.-H., Kim K.Y. (2021). Research progress on the identification and pharmacological activity of the active components of the *Rhododendron* species. Bosci. Biotechnol. Res. Asia.

[4] Olennikov D.N., Nikolaev V.M., Chirikova N.K. (2021). Sagan dalya tea, a new “old” probable adaptogenic drug: Metabolic characterization and bioactivity potentials of *Rhododendron adamsii* leaves. Antioxidants.

[5] Olennikov D.N., Kashchenko N.I., Chirikova N.K. (2026). *Rhododendron adamsii* flowers as a potential source of tea-derived flavonoid antioxidants. Horticulturae.

[6] Olennikov D.N., Kirillina C.S., Chirikova N.K. (2021). Water-soluble melanoidin pigment as a new antioxidant component of fermented willowherb leaves (*Epilobium angustifolium*). Antioxidants.

[7] Olennikov D.N., Kashchenko N.I., Chirikova N.K. (2017). Meadowsweet teas as new functional beverages: Comparative analysis of nutrients, phytochemicals and biological effects of four *Filipendula* species. Molecules.

[8] Kashchenko N.I., Olennikov D.N., Chirikova N.K. (2021). Metabolites of Siberian raspberries: LC-MS profile, seasonal variation, antioxidant activity, and thermal stability of *Rubus matsumuranus* phenolome. Plants.

[9] Olennikov D.N., Chemposov V.V., Chirikova N.K. (2021). Metabolites of prickly rose: Chemodiversity and digestive-enzyme-inhibiting potential of *Rosa acicularis* and the main ellagitannin rugosin D. Plants.

[10] Olennikov D.N., Khandy M.T., Chirikova N.K. (2022). Oriental strawberry metabolites: LC–MS profiling, antioxidant potential, and postharvest changes of *Fragaria orientalis* fruits. Horticulturae.

[11] Kukina T.P., Elshin I.A., Sal’nikova O.I., Kolosov P.V., Sandag C., Karakai D.A., Bondareva M.A., Nefedov A.A., Chirkova V.Y., Sharlaeva E.A. (2023). Composition of lipophilic components of *Rhododendron adamsii* Rehd. ether extract and activity against SARS-CoV-2 main protease. Russ. J. Bioorg. Chem..

[12] Kanojia A., Shrestha D.K., Dijkwel P.P. (2021). Primary metabolic processes as drivers of leaf ageing. Cell. Mol. Life Sci..

[13] Chacón D.I., Riley-Saldaña C.A., González-Esquinca A.R. (2012). Secondary metabolites during early development in plants. Phytochem. Rev..

[14] Song R., Kelman D., Johns K.L., Wright A.D. (2012). Correlation between leaf age, shade levels, and characteristic beneficial natural constituents of tea (*Camellia sinensis*) grown in Hawaii. Food Chem..

[15] Chan E.W.C., Lim Y.Y., Chew Y.L. (2007). Antioxidant activity of *Camellia sinensis* leaves and tea from a lowland plantation in Malaysia. Food Chem..

[16] Murdiono W.E., Salman N.A.S., Razak N.A.A., Halmi M.I.E., Yong J.W.H., Jalil A.W.M., Burslem D.F.R.P., Mahmud K. (2025). Effects of leaf ages, altitude and clone types on nutrient elements and antioxidant activity of tea (*Camellia sinensis* L. (O) Kuntze) in tropical conditions. Appl. Food Res..

[17] Hosoya T., Kubota M., Kumazawa S. (2024). Characterization and antioxidant activity of green tea (*Camellia sinensis*) using ^1^H NMR-based metabolomics. Food Sci. Technol. Res..

[18] Zhang M., Wang Y., Li Q., Wang Q., Ma S., Jia X., Zhang Q., Ye J. (2026). Influence of tea stand age on leaf biochemical components, mineral elements, and rhizosphere soil properties in Wuyi Rougui (*Camellia sinensis* (L.) Kuntze). Horticulturae.

[19] Chernonosov A.A., Karpova E.A., Karakulov A.V. (2023). Metabolomic profiling of three *Rhododendron* species from Eastern Siberia by liquid chromatography with high-resolution mass spectrometry. S. Afr. J. Bot..

[20] Kukina T.P., Elshin I.A., Sal’nikova O.I., Belenkaya S.V., Kolosova E.A., Volosnikova E.A., Shchegolkova V.O., Shcherbakov D.N. (2026). How to turn a poisonous plant into medicine: Non-polar extracts of *Rhododendron adamsii* (sagan dalya) are free of grayanotoxins and inhibit the SARS-CoV-2 main protease. Molecules.

[21] Olennikov D.N., Kashchenko N.I. (2026). Natural variation in leaf flavonoids among Siberian populations of *Rhododendron adamsii* (Ericaceae) and the identification of new acylated myricetin glycosides. Russ. J. Plant Physiol..

[22] Olennikov D.N., Kashchenko N.I. (2026). New flavonoids from *Rhododendron adamsii*. I. Myricetin glycosides. Chem. Nat. Comp..

[23] Dashiev D.B. (2001). Ddud Rtsi (The Canon of Tibetan Medicine).

[24] Subramaniyan V., Hanim Y.U. (2025). Role of pancreatic lipase inhibition in obesity treatment: Mechanisms and challenges towards current insights and future directions. Int. J. Obes..

[25] Uuh Narvaez J.J., Chan Zapata I., Segura Campos M.R. (2025). Combined potential of orlistat with natural sources and their bioactive compounds against obesity: A review. Molecules.

[26] Buchholz T., Melzig M.F. (2015). Polyphenolic compounds as pancreatic lipase inhibitors. Planta Med..

[27] Rocha S., Proença C., Araújo A.N., Freitas M., Rufino I., Aniceto N., Silva A.M.S., Carvalho F., Guedes R.C., Fernandes E. (2025). Flavonoids as potential modulators of pancreatic lipase catalytic activity. Pharmaceutics.

[28] Nasri R., Bidel L.P.R., Rugani N., Perrier V., Carrière F., Dubreucq E., Jay-Allemand C. (2019). Inhibition of CpLIP2 lipase hydrolytic activity by four flavonols (galangin, kaempferol, quercetin, myricetin) compared to orlistat and their binding mechanisms studied by quenching of fluorescence. Molecules.

[29] Welch K.D., Panter K.E., Gardner D.R. (2012). The good and the bad of poisonous plants: An introduction to the USDA-ARS poisonous plant research laboratory. J. Med. Toxicol..

[30] Belova N.V. (1970). Andromedotoxin in rhododendrons. Chem. Nat. Compd..

[31] Banti C.N., Hadjikakou S.K. (2021). Evaluation of toxicity with brine shrimp assay. Bio Protoc..

[32] Waghulde S., Kale M.K., Patil V. (2019). Brine shrimp lethality assay of the aqueous and ethanolic extracts of the selected species of medicinal plants. Proceedings.

[33] Olennikov D.N., Kashchenko N.I. (2023). Acylated flavonoids from *Cucumis sativus* inhibit the activity of human pancreatic lipase. Appl. Biochem. Microbiol..

[34] Olennikov D.N., Kashchenko N.I. (2023). New inhibitors of pancreatic α-amylase from *Rhaponticum uniflorum*. Appl. Biochem. Microbiol..

[35] Hamidi M.R., Jovanova B., Panovska T.K. (2014). Toxicological evaluation of the plant products using Brine Shrimp (*Artemia salina* L.) model. Maced. Pharm. Bull..

[36] Postelnicu T., Lovric M. (2014). Probit Analysis. International Encyclopedia of Statistical Science.

[37] Schymanski E.L., Jeon J., Gulde R., Fenner K., Ruff M., Singer H.P., Hollender J. (2014). Identifying small molecules via high resolution mass spectrometry: Communicating confidence. Environ. Sci. Technol..

[38] Bellumori M., Pallecchi M., Zonfrillo B., Lucio L., Menicatti M., Innocenti M., Mulinacci N., Bartolucci G. (2023). Study of mono and di-*O*-caffeoylquinic acid isomers in *Acmella oleracea* extracts by HPLC-MS/MS and application of linear equation of deconvolution analysis algorithm for their characterization. Pharmaceuticals.

[39] Miklavčič Višnjevec A., Baker P.W., Peeters K., Schwarzkopf M., Krienke D., Charlton A. (2021). HPLC-DAD-qTOF compositional analysis of the phenolic compounds present in crude tomato protein extracts derived from food processing. Molecules.

[40] Cao H., Chen X. (2012). Structures required of flavonoids for inhibiting digestive enzymes. Anticancer Agents Med. Chem..

[41] Castillo-Luna A., Miho H., Ledesma-Escobar C.A., Priego-Capote F. (2023). Comparison of drying techniques for extraction of bioactive compounds from olive-tree materials. Foods.

[42] Koo S.I., Noh S.K. (2007). Green tea as inhibitor of the intestinal absorption of lipids: Potential mechanism for its lipid-lowering effect. J. Nutr. Biochem..

[43] Glisan S.L., Grove K.A., Yennawar N.H., Lambert J.D. (2016). Inhibition of pancreatic lipase by black tea theaflavins: Comparative enzymology and in silico modeling studies. Food Chem..

[44] Tang Z., Zhang Q. (2021). The potential toxic side effects of flavonoids. Biocell.

[45] Meyer B.N., Ferrigni N.R., Putnam J.E., Jacobsen L.B., Nichols D.E., McLaughlin J.L. (1982). Brine Shrimp: A convenient general bioassay for active plant constituents. Planta Med..

[46] Fraiser L.H., Kanekal S., Kehrer J.P. (1991). Cyclophosphamide toxicity. Characterising and avoiding the problem. Drugs.

[47] Lee W., Wang Y.C. (2015). Assessing developmental toxicity of caffeine and sweeteners in medaka (*Oryzias latipes*). Springerplus.

[48] Coe F.G., Parikh D.M., Johnson C.A. (2010). Alkaloid presence and brine shrimp (*Artemia salina*) bioassay of medicinal species of eastern Nicaragua. Pharm. Biol..

[49] Ntungwe E., Domínguez-Martín E.M., Roberto A., Tavares J., Isca V.M.S., Pereira P., Cebola M.-J., Rijo P. (2020). *Artemia* species: An important tool to screen general toxicity samples. Curr. Pharm. Des..

[50] Surya K.G., Shanmugam R., Behera A., Jothinathan M.K.D. (2024). Assessment of antibacterial properties and cytotoxic effect of ethanolic extracts of *Clitoria ternatea* and *Camellia sinensis* herbal formulation against clinical pathogens. Cureus.

[51] Konan A., Golly K., Kra A., Adima A., Lohoues E. (2022). Phytochemical screening and toxicity assessment of *Imperata cylindrica* (L.) P. Beauv. (Poaceae) raw extracts with brine shrimp (*Artemia salina*) lethality assay. J. Biosci. Med..

[52] Bajatovska A.M., Zhivikj Z., Shutevska K., Karapandzova M., Petreska Ivanovska T., Petrushevska-Tozi L., Kadifikova Panovska T. (2023). Toxicity screening of weight loss tea products by Brine Shrimp Lethality Assay. Maced. Pharm. Bull..

[53] Vengrytė M., Raudonė L. (2026). Seasonal dynamics of leaf phenolic compounds in *Rhododendron tomentosum* Harmaja growing in a coniferous swamp forest. Plants.

[54] Rathore N., Thakur D., Chawla A. (2018). Seasonal variations coupled with elevation gradient drives significant changes in eco-physiological and biogeochemical traits of a high altitude evergreen broadleaf shrub, *Rhododendron anthopogon*. Plant Physiol. Biochem..

[55] Lv X., Hao J., Zhao Y., Li C., Quan W. (2023). Seasonal variations of low-molecular-weight organic acids in three evergreen broadleaf *Rhododendron* forests. Metabolites.

[56] Liu B., Xia Y., Krebs S.L., Medeiros J., Arora R. (2019). Seasonal responses to cold and light stresses by two elevational ecotypes of *Rhododendron catawbiense*: A comparative study of overwintering strategies. Environ. Exp. Bot..

[57] Li H., Guo Q., Yang L., Quan H., Wang S. (2022). Seasonal eco-physiology characteristics of four evergreen *Rhododendron* species to the subalpine habitats. Forests.

[58] Bitter J., Pfeiffer M., Borg A.J.E. (2023). Enzymatic β-elimination in natural product *O*- and *C*-glycoside deglycosylation. Nat. Commun..

[59] Xu J., Deng X., Wu Y., Zhou M., Du C., Wang Q., Xia Y., He J., Yuan W., Wu W. (2025). Characteristic changes and potential markers of flavour in raw pu-erh tea with different ageing cycles analysed by HPLC, HS-SPME-GC-MS, and OAV. Foods.

[60] Klepacka J. (2022). Tea infusions as a source of phenolic compounds in the human diet. Appl. Sci..

[61] Kodama D.H.G., Lajolo A.E.S.S., Genovese F.M., Inés M. (2010). Flavonoids, total phenolics and antioxidant capacity: Comparison between commercial green tea preparations. Food Sci. Technol..

[62] Komes D., Horžić D., Belščak A., Ganić K.K., Vulić I. (2020). Green tea preparation and its influence on the content of bioactive compounds. Food Res. Int..

[63] Zhou J., Wang W., Yin Z., Zheng G., Chen J., Li J., Chen L., Zhang Q. (2021). Quercetin is a promising pancreatic lipase inhibitor in reducing fat absorption in vivo. Food Biosci..

[64] Bustos A.-S., Håkansson A., Linares-Pastén J.A., Nilsson L. (2020). Interaction between myricetin aggregates and lipase under simplified intestinal conditions. Foods.

[65] Brimson J.M., Prasanth M.I., Kumaree K.K., Thitilertdecha P., Malar D.S., Tencomnao T., Prasansuklab A. (2023). Tea plant (*Camellia sinensis*): A current update on use in diabetes, obesity, and cardiovascular disease. Nutrients.

[66] Li Y.F., Chang Y.Q., Deng J. (2016). Prediction and evaluation of the lipase inhibitory activities of tea polyphenols with 3D-QSAR models. Sci. Rep..

[67] Yuda N., Tanaka M., Suzuki M., Asano Y., Ochi H., Iwatsuki K. (2012). Polyphenols extracted from black tea (*Camellia sinensis*) residue by hot-compressed water and their inhibitory effect on pancreatic lipase in vitro. J. Food Sci..

[68] Vieira-Almeida E.C., Pigoso A.A., de Almeida A.F. (2015). *Camellia sinensis* extract inhibits in vitro pancreatic lipase and has preventive effect on obesity in female rat fed a high-fat diet. Afr. J. Pharm. Pharmacol..

[69] Qin X.-Y., Hou X.-D., Zhu G.-H., Xiong Y., Song Y.-Q., Zhu L., Zhao D.-F., Jia S.-N., Hou J., Tang H. (2022). Discovery and characterization of the naturally occurring inhibitors against human pancreatic lipase in *Ampelopsis grossedentata*. Front. Nutr..

[70] Ma L., Hou X., Qin X., He R., Yu H., Hu Q., Guan X., Jia J., Hou J., Lei T. (2022). Discovery of human pancreatic lipase inhibitors from root of *Rhodiola crenulata* via integrating bioactivity-guided fractionation, chemical profiling and biochemical assay. J. Pharm. Anal..

[71] Harborne J.B., Williams C.A. (1973). A chemotaxonomic survey of flavonoids and simple phenols in leaves of the Ericaceae. Bot. J. Linn. Soc..

[72] Frenț O.-D., Stefan L., Morgovan C.M., Duteanu N., Dejeu I.L., Marian E., Vicaș L., Manole F. (2024). A systematic review: Quercetin—Secondary metabolite of the flavonol class, with multiple health benefits and low bioavailability. Int. J. Mol. Sci..

[73] Buckingham J., Munasinghe V.R.N. (2015). Dictionary of Flavonoids.

[74] Khaidav T., Altanchimeg B., Varlamova T.S. (1985). Medicinal Plants in Mongolian Medicine.

[75] Bedrood Z., Rameshrad M., Hosseinzadeh H. (2018). Toxicological effects of *Camellia sinensis* (green tea): A review. Phytother. Res..

[76] Lechtenberg M., Dierks F., Sendker J., Louis A., Schepker H., Hensel A. (2014). Extracts from *Rhododendron ferrugineum* do not exhibit grayanotoxin I: An analytical survey on grayanotoxin I within the genus *Rhododendron*. Planta Med..

[77] Dursun I., Kanbur E.D., Kara Y., Kolaylı S. (2025). Determination of grayanotoxin-III amount in the rhododendron honey and flowers samples using UHPLC-Orbitrap^®^-HRMS. Toxicon.

[78] Paudel B., Bhattarai H.D., Kim I.C. (2014). Estimation of antioxidant, antimicrobial activity and brine shrimp toxicity of plants collected from Oymyakon region of the Republic of Sakha (Yakutia), Russia. Biol. Res..

[79] Karchesy Y.M., Kelsey R.G., Constantine G. (2016). Biological screening of selected Pacific Northwest forest plants using the brine shrimp (*Artemia salina*) toxicity bioassay. SpringerPlus.

